# Identification of a conserved neutralizing epitope on the ASFV H240R protein by a nanobody that inhibits viral attachment

**DOI:** 10.1016/j.jbc.2026.113470

**Published:** 2026-08-19

**Authors:** Dian Jiao, Binbin Ren, Dongying Liu, Yue Wang, Danyang Zhang, Jianan Tao, Zhanning Shen, Xuwen Sun, Hui Zhou, Zixiang Zhu, Julian A. Hiscox, James P. Stewart, Qin Zhao, Yani Sun

**Affiliations:** 1 College of Veterinary Medicine, Northwest A&F University, Yangling, China; 2 Engineering Research Center of Efficient New Vaccines for Animals, Ministry of Education, Yangling, China; 3 Key Laboratory of Ruminant Disease Prevention and Control (West), Ministry of Agriculture and Rural Affairs, Yangling, China; 4 Engineering Research Center of Efficient New Vaccines for Animals, Universities of Shaanxi Province, Yangling, China; 5 State Key Laboratory for Animal Disease Control and Prevention, College of Veterinary Medicine, Lanzhou University, Lanzhou Veterinary Research Institute, Chinese Academy of Agricultural Sciences, Lanzhou, China; 6 Department of Infection Biology and Microbiomes, Institute of Infection, Veterinary and Ecological Sciences, University of Liverpool, Liverpool, United Kingdom

**Keywords:** ASFV, neutralizing antibodies, H240R protein, nanobody, infection, virus entry, epitope mapping

## Abstract

African swine fever virus (ASFV), a highly contagious double-stranded DNA virus, causes a hemorrhagic disease with mortality approaching 100% in domestic pigs. Despite intensive efforts, effective vaccine options remain limited, highlighting the need for continuously identifying viral neutralizing epitopes. Nanobodies can effectively engage cryptic epitopes that are often inaccessible to conventional antibodies due to their small size. Here, we immunized a Bactrian camel with recombinant ASFV H240R expressed in a bacterial system, and an H240R-specific VHH phage-display library was constructed and screened, yielding a nanobody named H240R-Nb82. H240R-Nb82 efficiently neutralized distinct ASFV isolates (genotypes Ⅰ, Ⅱ, and their recombinants) in porcine alveolar macrophages. Mechanistically, H240R-Nb82 inhibited the early stage of ASFV infection by blocking viral attachment to host cells. Epitope mapping using truncation analysis and site-directed alanine mutagenesis showed that H240R-Nb82 binds to a linear, native virion-accessible conserved region within H240R (residues ^33^GHYPFSFELK^42^) and His34 made an important contribution to H240R-Nb82 binding. H240R-Nb82 also directly bound purified ASFV virions. Moreover, immunization of mice with the epitope-containing antigen induced sera capable of neutralizing ASFV infection in porcine alveolar macrophages, demonstrating the protective potential of this conserved determinant. Collectively, these findings define a previously unrecognized conserved neutralizing epitope on ASFV H240R and provide a new target protein for developing ASFV subunit vaccines.

African swine fever virus (ASFV) is a large, structurally complex DNA virus that causes African swine fever (1). The outbreaks have resulted in sustained and significant losses to the global swine industry (2). However, the safe and broadly effective commercial vaccines are scarcely available, a core obstacle being that the protective immune mechanisms against the virus are not fully understood (3). Previous studies have indicated that the traditional inactivated vaccines fail to provide protection, seemingly suggesting a difficulty in eliciting effective neutralizing antibodies (4).

ASFV possesses a large genome encoding approximately 150 to 200 proteins from 161 to 167 open reading frames (5). The mature virion is characterized by a complex, multilayered architecture comprising a nucleoid, core shell, inner envelope, capsid, and outer envelope (6). While this overall structural framework is established, the specific functions of most ASFV-encoded proteins, particularly those constituting the virion, remain poorly defined (7). Elucidating these roles is crucial for advancing rational strategies in ASFV vaccine and diagnostic development (8).

In general, proteins located on the outer envelope are considered particularly important for understanding viral attachment, entry, and antibody-mediated neutralization, because they are directly exposed to the extracellular environment and are readily accessible to host antibodies or other binding molecules (9). However, ASFV infectivity is not necessarily restricted to virions with a fully intact outer envelope. Previous studies have suggested that infectious ASFV particles may include virions lacking the outer envelope or particles whose outer envelope is disrupted during viral entry, and these particles can still retain infectivity (10). Therefore, under certain infection-related conditions or in specific virion states, proteins located beneath the outer envelope, particularly those constituting the viral capsid, may become exposed and accessible to neutralizing antibodies or other antiviral molecules. Functional characterization of these capsid-associated proteins is thus important for a more comprehensive understanding of ASFV neutralizing or antibody-accessible epitopes.

The icosahedral capsid, a key protective layer beneath the outer envelope, is assembled from 17,280 protein subunits organized into pentasymmetrons and trisymmetrons (9). This assembly includes the major capsid protein p72 and several minor capsid-associated proteins, such as M1249L, p17, p49, and H240R. Among these, p72, p49, and p17 have been implicated in outer capsid formation (11). Functionally, H240R interacts with p72 and is essential for proper virion morphogenesis (12). Beyond its structural role, H240R also acts as a virulence factor by antagonizing the host cGAS-STING-mediated type I interferon signaling pathway (13). Although the accessibility of H240R on intact enveloped ASFV virions remains uncertain, its potential exposure in envelope-deficient or entry-associated virion states suggests that H240R may represent a biologically relevant target for investigating ASFV capsid-associated antibody-accessible epitopes.

The identification and characterization of defined antigenic epitopes is a critical foundation for developing effective viral subunit vaccines (14). Previously, some ASFV proteins such as p30, p54, and p72 have been shown to only elicit partially protective immune responses in pigs, and the presence of neutralizing epitopes in these proteins was characterized (15). However, few studies have characterized neutralizing epitopes in other ASFV proteins, such as H240R protein. Additionally, to date, the identification of ASFV epitopes has mostly relied on conventional monoclonal antibodies or peptide scanning using immune sera (16). Nanobodies are derived from the variable domains of camelid heavy-chain antibodies. Owing to their small size, they can recognize epitopes located in concave antigenic regions (17). Therefore, nanobodies may serve as tools for identifying a broader range of more cryptic antigenic epitopes (18). In this study, we generated a nanobody library by immunizing a Bactrian camel with recombinant H240R protein. Subsequently, a neutralizing nanobody that targets a highly conserved region of H240R was identified and effectively blocked viral attachment. Notably, this represents the first identification of a neutralizing epitope on the H240R protein. Our findings not only advance the understanding of ASFV antigenicity but also provide a defined, conserved epitope that serves as a promising candidate for the development of subunit vaccines.

## Results

### Preparation of nanobody against ASFV H240R

**Figure 1:**
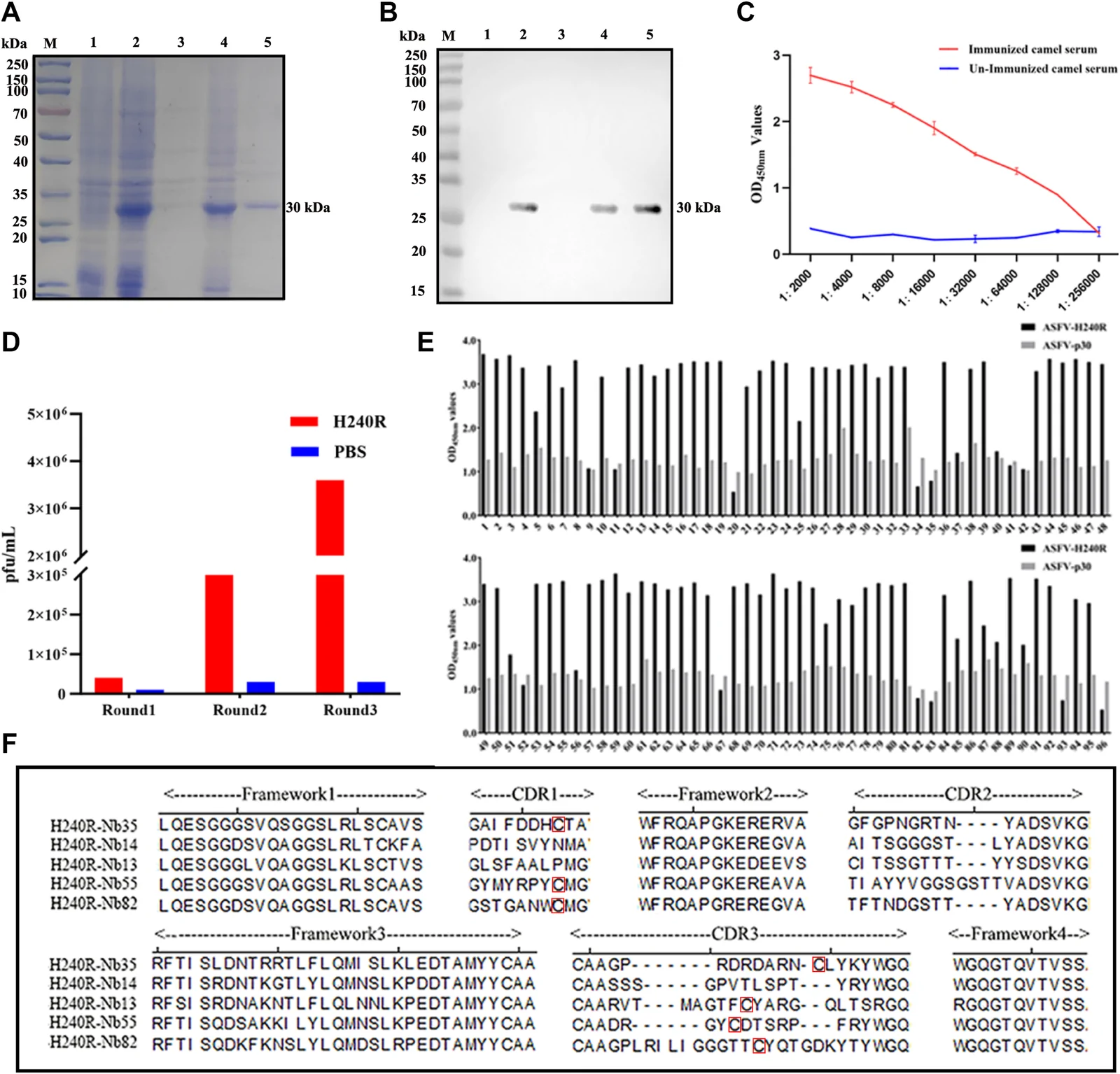
**Preparation of nanobodies against ASFV H240R.***A*, SDS-PAGE analysis of the recombinant H240R protein expressed in *E. coli* BL21 (DE3). *B*, Western blotting analysis of the recombinant H240R protein reaction with convalescent porcine sera from ASFV-infected pigs as the primary antibody. M: protein molecular weight marker; lane 1: uninduced whole bacterial lysate; lane 2: induced whole bacterial lysate; lane 3: supernatant after sonication; lane 4: pellet after sonication; lane 5: purified H240R protein. *C*, the titers of immunized camel sera against H240R using the indirect ELISA with recombinant H240R as coating antigen. Error bars indicate the mean ± SD, n = 3. *D*, phage display enrichment after three rounds of biopanning. Negative control: PBS. *E*, screening of 96 clones after the third round of biopanning using the indirect ELISA with H240R as coating antigen, with p30 serving as the negative control antigen. Cutoff value was defined as: *A*_*4*50nm_ (sample)/*A*_450nm_ (negative control) ≥ 3. Negative control: ASFV-p30 expressed with the same system and conditions as the recombinant H240R. *F*, sequence alignment of anti-H240R nanobodies. Cysteine residues within CDR1 and CDR3 are highlighted with *red boxes*. ASFV, African swine fever virus.

First, the ASFV H240R protein was expressed using a prokaryotic expression system in *Escherichia coli* BL21 (DE3) strain and purified for camel immunization and nanobody screening. SDS-PAGE analysis showed that a distinct band was observed at approximately 30 kDa, consistent with the predicted molecular weight of H240R (Fig. 1*A*). To further evaluate the homogeneity and aggregation status of the antigen used for immunization, the purified H240R protein was analyzed by size-exclusion chromatography (SEC). The protein eluted predominantly as a single major peak, and SDS-PAGE analysis of the corresponding fraction confirmed the presence of H240R, suggesting that the antigen preparation was largely homogeneous and showed no substantial aggregation under the conditions tested (Fig. S5). Western blotting analysis showed that probing with convalescent porcine sera from ASFV-infected pigs yielded a strong, specific signal at the same position (Fig. 1*B*), while no reactivity was detected in the negative-control pig sera (data not shown). These results indicated the successful expression and purification of recombinant ASFV H240R protein with retained antigenicity.

Then, the recombinant H240R protein was used as the antigen for camel immunization and nanobodies screening. A schematic overview of the screening workflow and neutralization testing is shown (Fig. S1). Following five immunizations, the camel exhibited a strong humoral immune response, with endpoint ELISA titer against H240R of 1:256,000 (Fig. 1*C*). A phage-display VHH library was constructed from peripheral blood lymphocytes, yielding approximately 2 × 10^8^ independent transformants. PCR screening of 48 randomly selected clones revealed inserts of the expected size in 46 clones, corresponding to a 95.8% insert-positive rate (Fig. S2). Sequencing confirmed that each positive clone contained a unique complementarity-determining region 3 (CDR3) sequence, with pairwise amino acid identities below 50%, indicating a high library diversity (data not shown).

Three rounds of biopanning were performed using the purified H240R as the capture antigen and phosphate buffer (PBS) as a negative control. A significant enrichment of H240R-binding phages was observed, far exceeding the negative control (Fig. 1*D*). Periplasmic extracts from 96 individual clones after the third panning round were tested by indirect ELISA. Twelve clones, with *A*_450nm_ values exceeding the negative control (ASFV-p30 expressed with the same systems and conditions as H240R) by more than threefold, were considered as positive reactivity with H240R (Fig. 1*E*). Sequencing results showed that five unique anti-H240R nanobodies were obtained and designated H240R-Nb13, -Nb14, -Nb35, -Nb55, and -Nb82 (Fig. 1*F*).

### Screening of nanobodies binding to natively folded H240R protein

**Figure 2:**
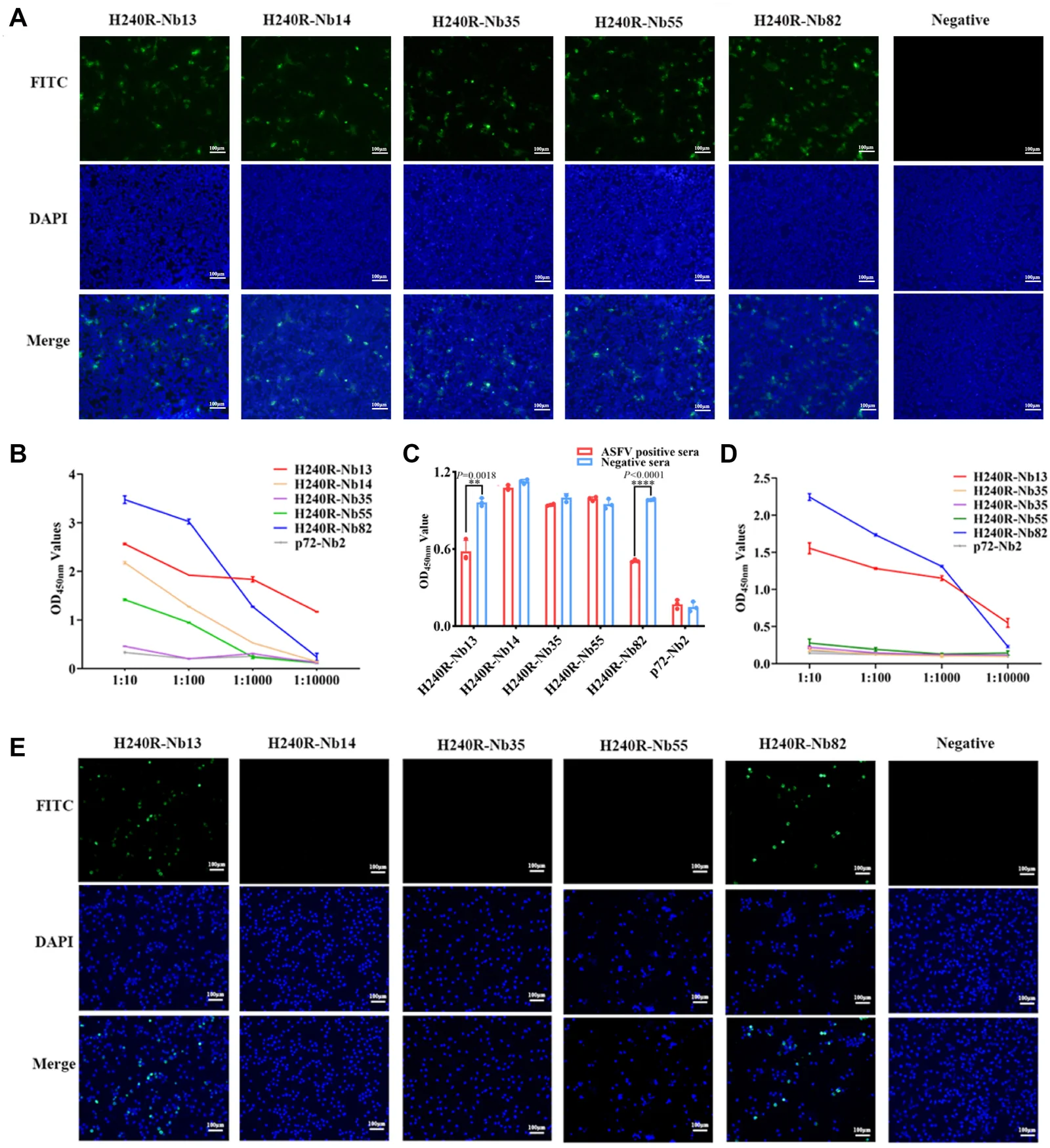
**Screening of nanobodies binding to the natively folded H240R protein.***A*, intracellular expression of H240R-specific nanobodies fused to HRP (nanobody-HRP) in HEK293T cells by IFA. Nanobody-HRP expression was visualized with FITC (*green*), and nuclei were counterstained with DAPI (*blue*). Negative: the blank vector transfected into the HEK293T cells. Scale bar: 100 μm. *B*, antigen-binding capability of nanobody-HRP fusion proteins (from 1:10–1:10,000) by the direct ELISA coated with the recombinant H240R protein expressed in bacterial system. Negative control: p72-Nb2. Error bars indicate the mean ± SD, n = 3. *C*, detection of ASFV-positive pig sera for blocking the nanobodies-HRP fusion proteins reaction with the H240R. A lower *A*_450nm_ value indicates an inversely proportional increase in the blocking effect of the ASFV-positive pig sera. The lower the *A*_450nm_ value than the negative sera showed the stronger blocking effect of the ASFV-positive sera. Negative control: p72-Nb2. The data shown are representative of three replicates (two-tailed paired Student’s *t* test). Error bars indicate the mean ± SD, n = 3. *D and E*, detection of nanobodies-HRP fusion proteins binding to the purified ASFV virions by direct ELISA with p72-Nb2 as the irrelevant control. Error bars indicate the mean ± SD, n = 3 (*D*). For IFA (*E*), cells were infected with ASFV and mixed with nanobodies, which was subsequently visualized with an anti-His FITC-conjugated antibody. The negative control was the supernatant from HEK293T cells transfected with the empty vector, processed under identical incubation and detection conditions. Scale bar: 100 μm. ASFV, African swine fever virus; HRP, horseradish peroxidase; IFA, immunofluorescence assay.

H240R-specific nanobodies C-terminally fused to horseradish peroxidase (HRP) were generated in human embryonic kidney 293T (HEK293T) cells as described previously (19). Expression of all five nanobody-HRP fusion proteins in transfected cells was confirmed by indirect immunofluorescence assay (IFA) (Fig. 2*A*). As these constructs contained a secretion signal peptide, the fusion proteins were then secreted into the culture supernatant and collected for subsequent experiments. Then, direct ELISA using recombinant H240R-coated plates demonstrated that these nanobodies retained both antigen-binding capability and HRP activity, with H240R-Nb13 and H240R-Nb82 exhibiting higher apparent binding signals than H240R-Nb14 and H240R-Nb55 (Fig. 2*B*). To investigate whether these epitopes recognized by the five nanobodies can induce immune responses in the ASFV-infected pigs, a competitive ELISA was conducted. The results showed that the H240R-Nb13 and -Nb82 could be blocked by the ASFV-positive pig sera for binding to H240R, and H240R-Nb82 displayed slightly higher competitive efficiency than H240R-Nb13 (Fig. 2*C*). To assess nanobody-specific binding to H240R of native state, ASFV virions were purified *via* sucrose density gradient centrifugation (Fig. S3). Virions were collected from the 25% to 56% sucrose region of the gradient (11). Using a direct ELISA coating with ASFV virions, the results demonstrated that the purified ASFV particles exhibited clear binding activity with both H240R-Nb13 and H240R-Nb82 (Fig. 2*D*). Finally, we evaluated the binding of these fusion proteins to H240R in ASFV (genotype Ⅱ ASFV CN/GS/2018 isolate)-infected porcine alveolar macrophages (PAMs). The IFA was performed using the five nanobodies-HRP fusion proteins as primary antibody, respectively, and the results demonstrated that the H240R-Nb13 and -Nb82 also specifically bound to ASFV (Fig. 2*E*), supporting their ability to recognize H240R in the context of viral infection.

These results demonstrated that H240R-Nb13 and -Nb82 recognize the H240R protein in its native conformation, and epitopes recognized by them can induce immune responses after ASFV-infected pigs.

### Identification of neutralizing nanobodies against ASFV

**Figure 3:**
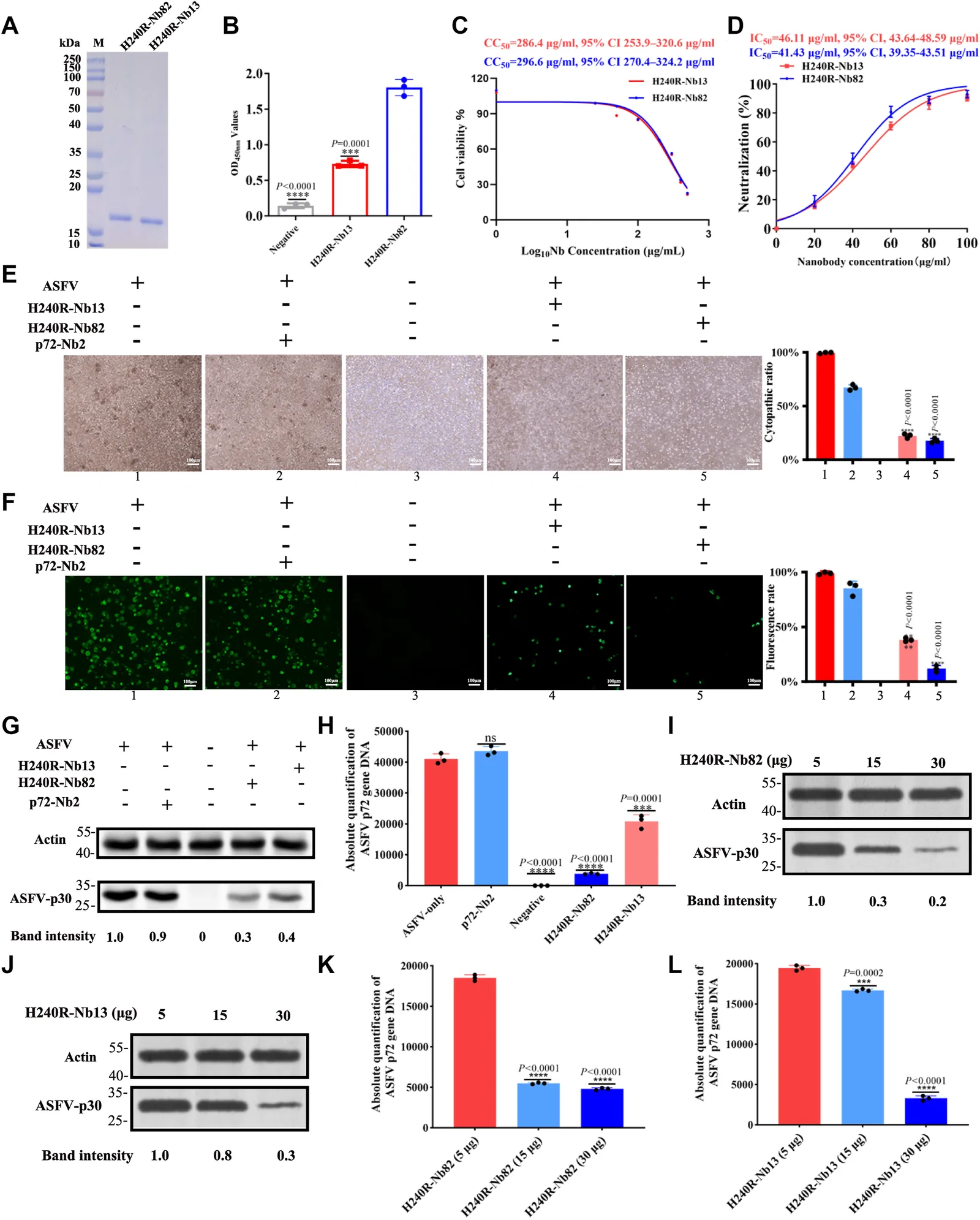
**Identification of neutralizing nanobodies against ASFV.***A*, SDS-PAGE analysis of prokaryotic expression and purification of monovalent H240R-Nb13 and -82. *B*, capture ELISA analysis of H240R-Nb13 and -82 binding to purified ASFV particles. The data shown are representative of three replicates (two-tailed paired Student’s *t* test). Error bars indicate the mean ± SD, n = 3. *C*, the CC_50_ analysis of H240R-Nb13 and -82 in PAMs. *D*, IC_50_ analysis of H240R-Nb13 and -82 against ASFV infection in PAMs. Error bars indicate the mean ± SD, n = 3. *E*, HAD assay to evaluate the neutralization effect of H240R-Nb13 and -82 against ASFV infection. The p72-Nb2 served as irrelevant nanobody control. Bright-field images were acquired with a light microscope and analyzed using ImageJ software. For each group, cells were plated in triplicate wells, and three fields of view per well were randomly selected for quantification. The bar chart shows the percentage of HAD-positive signals. Scale bar: 100 μm. *F*–*H*, analysis of the neutralization effect of H240R-Nb13 and -82 against ASFV infection using IFA (*F*), Western blotting (*G*), and qPCR (*H*). The p72-Nb2 served as irrelevant nanobody control. Fluorescence images were acquired with a fluorescence microscope and analyzed using ImageJ software. For each group, cells were plated in triplicate wells, and three fields of view per well were randomly selected for quantification. The bar chart represents the percentage of fluorescent positive signals. Scale bar: 100 μm. The data shown are representative of three replicates (one-way repeated-measures ANOVA followed by Dunnett’s multiple-comparisons test). Error bars indicate the mean ± SD, n = 3. *I*–*J*, Western blotting analysis of the dose-dependent neutralizing effect of H240R-Nb82 (*I*) and −13 (*J*) against ASFV infection. *K–L*, qPCR analysis of the dose-dependent neutralizing effect of H240R-Nb82 (*K*) and −13 (*L*) against ASFV infection. The data shown are representative of three replicates (one-way repeated-measures ANOVA followed by Dunnett’s multiple-comparisons test). Error bars indicate the mean ± SD, n = 3. ASFV, African swine fever virus; IFA, immunofluorescence assay; HAD, hemadsorption; PAMs, porcine alveolar macrophages.

Given that both H240R-Nb13 and -Nb82 can bind to the ASFV particles and the epitopes recognized by the nanobodies can induce immune responses in the pigs, we next evaluated whether these two H240R-specific nanobodies can neutralize ASFV infection in PAMs. First, H240R-Nb13 and -Nb82 were transformed into *E. coli* BL21 (DE3) strain. SDS-PAGE analysis confirmed that the two high-purity monovalent nanobodies were successfully expressed, as a single band of approximately 15 kDa (Fig. 3*A*). To further validate their binding capability, a capture ELISA with the nanobody as the coating antibody was performed. The results showed that both of the two nanobodies can capture the purified ASFV particles and the H240R-Nb82 possessed the higher virus-capturing activity (Fig. 3*B*). Before neutralization testing, the 50% cytotoxic concentration (CC_50_) values in PAMs were determined for H240R-Nb13 and -Nb82. The results showed that CC_50_ of the two nanobodies were 286.4 μg/ml and 296.6 μg/ml, respectively, indicating low cytotoxicity of them (Fig. 3*C*). Following, neutralization assays determined that the half-maximal inhibitory concentrations (IC_50_) of H240R-Nb13 and -Nb82 against ASFV (genotype Ⅱ ASFV CN/GS/2018 isolate) were 46.11 μg/ml and 41.43 μg/ml, respectively (Fig. 3*D*). Hemadsorption (HAD) is a characteristic phenotypic feature of ASFV infection in PAMs, in which porcine erythrocytes adhere to infected cells and form rosette-like structures, and is therefore commonly used as a phenotypic readout of ASFV infection. HAD assays revealed that H240R-Nb13 and -Nb82 were able to separately inhibit approximately 75% and 80% of the ASFV-induced HAD phenomenon compared with the ASFV-only and irrelevant p72-Nb2 controls (Fig. 3*E*). The IFA results also showed that compared with the ASFV-only and irrelevant p72-Nb2 control groups, the cells treated with H240R-Nb13 or H240R-Nb82 (30 μg) exhibited significantly reduced viral loads, with fluorescent signal intensity decreasing by approximately 60% and 80%, respectively (Fig. 3*F*). Western blotting analysis further showed a clear reduction in ASFV p30 protein expression in nanobody-treated groups compared to the ASFV-only and irrelevant p72-Nb2 controls, with H240R-Nb13 and -Nb82 reducing it by 50% and 70%, respectively (Fig. 3*G*). The qPCR results also demonstrated that both nanobodies significantly reduced the DNA levels of the p72 gene of the CN/GS/2018 strain, indicating H240R-Nb13 and -Nb82 exhibited neutralization rates of 50% and 85%, respectively (Fig. 3*H*).

Dose–response experiments demonstrated that H240R-Nb82 (30 μg and 15 μg) can effectively suppress replication of CN/GS/2018 (Fig. 3*I*), whereas H240R-Nb13 at 15 μg was insufficient to elicit effective inhibition (Fig. 3*J*). This dose-dependent effect was also directly validated by the qPCR data (Fig. 3, *K* and *L*).

Taken together, these findings indicate that H240R-Nb82 and -Nb13 are two effective and specific neutralizing nanobodies against CN/GS/2018, with H240R-Nb82 exhibiting stronger neutralizing activity.

### Neutralization against various ASFV strains of the H240R-Nb82

**Figure 4:**
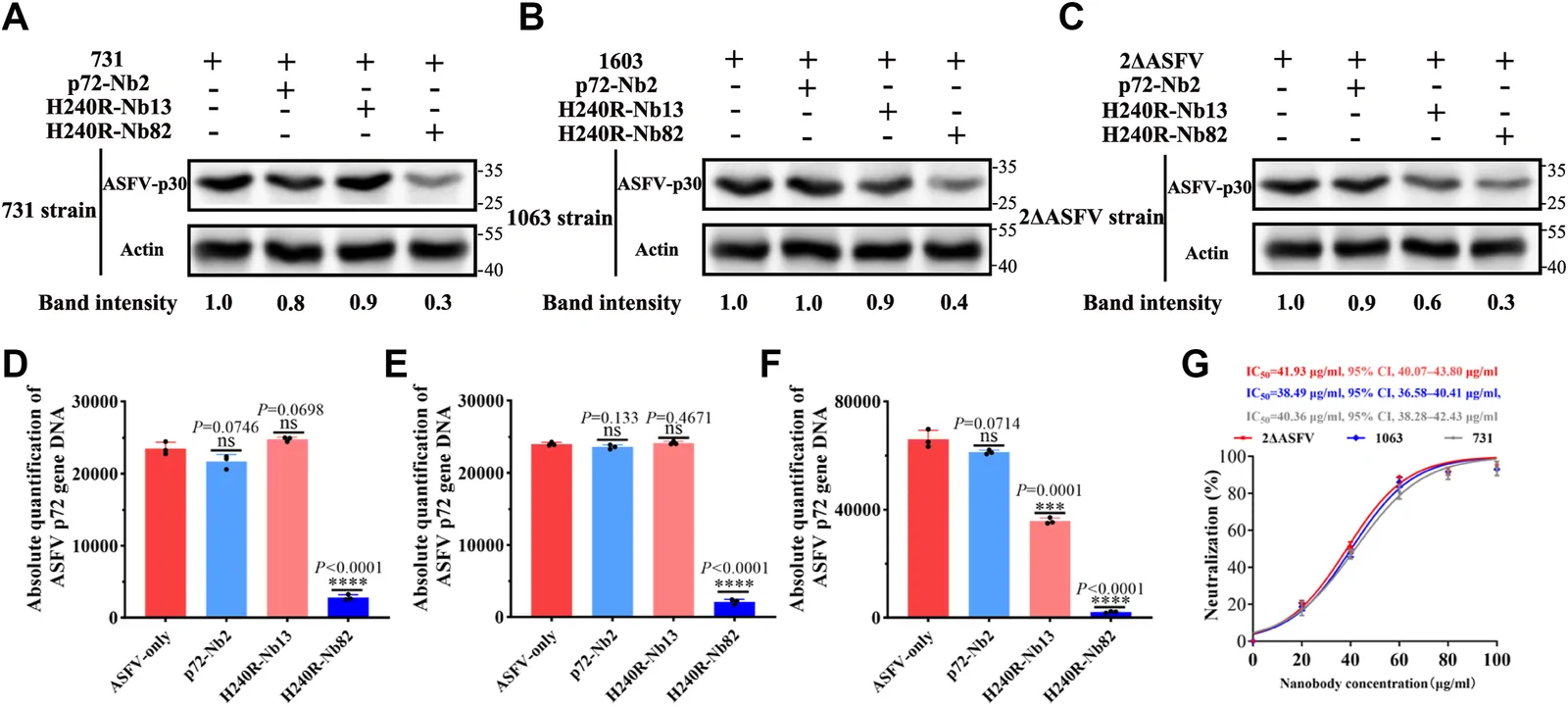
**Neutralization effects of H240R-Nb13 and -82 against different ASFV isolates in PAMs.***A*–*C*, analysis of the neutralizing effect of H240R-Nb13 and -82 against different ASFV isolates including 731, 1063, and 2ΔASFV by Western blotting. The p72-Nb2 served as irrelevant nanobody control. *D*–*F*, analysis of the neutralizing effect of H240R-Nb13 and -82 against different ASFV isolates including 731, 1063, and 2ΔASFV by qPCR. The p72-Nb2 served as irrelevant nanobody control. The data shown are representative of three replicates (one-way repeated-measures ANOVA followed by Dunnett’s multiple-comparisons test). Error bars indicate the mean ± SD, n = 3. ∗∗∗*p* < 0.001; ∗∗∗∗*p* < 0.0001. *G*, IC_50_ analysis of H240R-Nb82 against different ASFV isolates including strains 731, 1063, and 2ΔASFV in PAMs. Error bars indicate the mean ± SD, n = 3. ASFV, African swine fever virus; ns, not significant; PAMs, porcine alveolar macrophages.

To assess whether H240R-Nb13 and -Nb82 can neutralize other ASFV strains, we selected three genetically distinct ASFV isolates including a genotype I/II recombinant strain 731, a genotype I strain 1063, and a genotype II strain 2ΔASFV (two-gene deletion) for cross-neutralization profiling. Neutralization assays demonstrated that, compared with the ASFV-only and irrelevant nanobody p72-Nb2 controls, the H240R-Nb82 significantly reduced both expression of the viral p30 protein (Fig. 4, *A*–*C*) and the p72 viral DNA copies in PAMs infected with the three strains (Fig. 4, *D*–*F*). In contrast, H240R-Nb13 exhibited neutralizing activity only against the 2ΔASFV strain (Fig. 4, *C* and *F*), while no significant neutralizing effect was observed against the other two strains (Fig. 4, *A*, *B*, *D* and *E*). Notably, H240R-Nb82 showed potent inhibition against all tested strains, with IC_50_ values of 41.93, 38.49, and 40.36 μg/ml for 731, 2ΔASFV, and 1063, respectively (Fig. 4*G*). Under identical conditions, the irrelevant nanobody p72-Nb2 displayed no significant neutralizing activity. These results indicate that H240R-Nb82 could inhibit the replication of different genotypes ASFV strains. In contrast, H240R-Nb13 can only inhibit the replication of genotype Ⅱ ASFV strains.

### H240R-Nb82 blocks ASFV attachment to host cells

**Figure 5:**
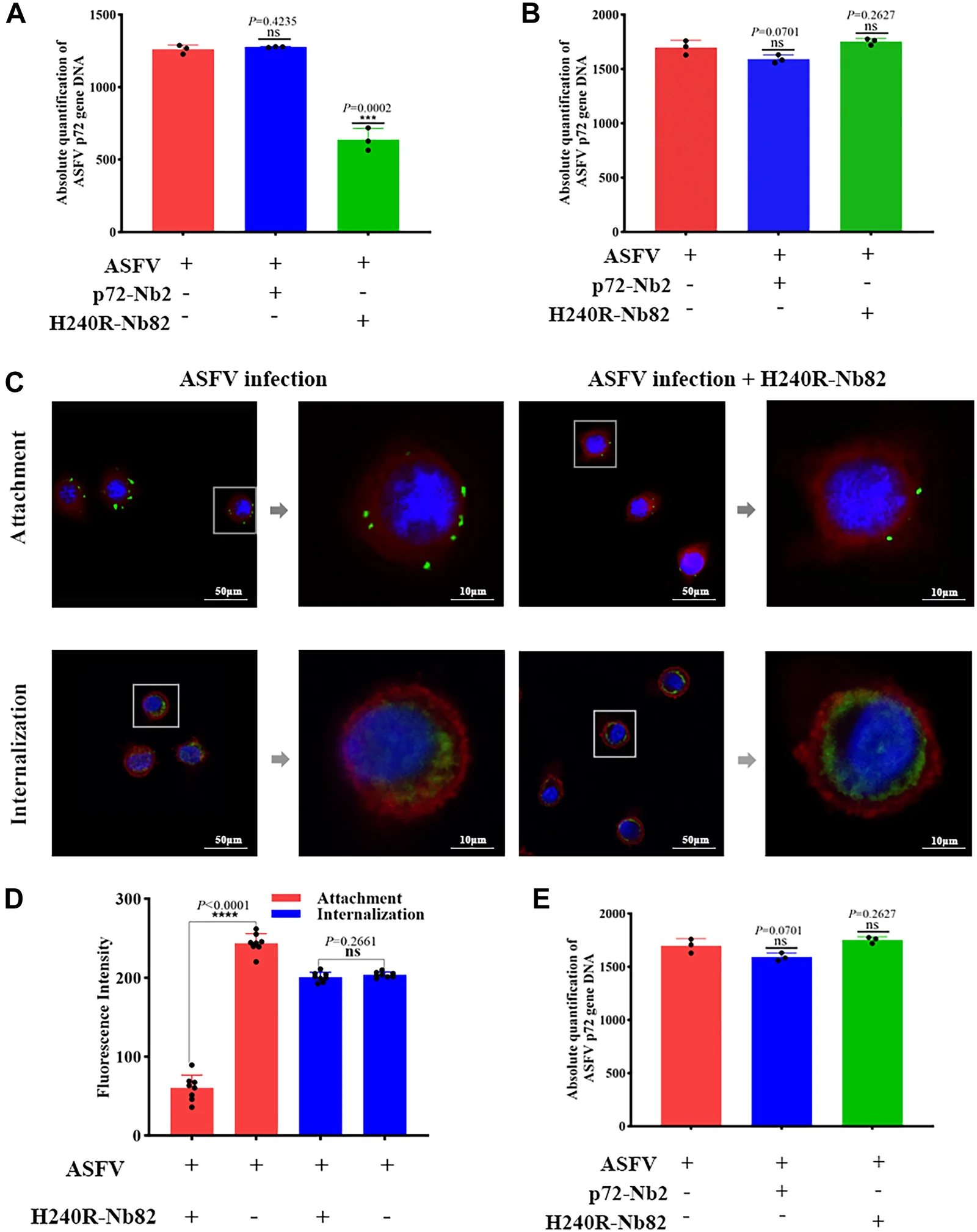
**The H240R-Nb82 inhibits ASFV attachment to host cells.***A*, qPCR analysis of H240R-Nb82 inhibiting ASFV attachment to PAMs. The p72-Nb2 served as irrelevant nanobody control. The data shown are representative of three replicates (two-tailed paired Student’s *t* test). Error bars indicate the mean ± SD, n = 3. ∗∗∗*p* < 0.001. *B*, qPCR analysis of H240R-Nb82 not inhibiting ASFV internalization. The p72-Nb2 served as irrelevant nanobody control. The data shown are representative of three replicates (two-tailed paired Student’s *t* test). Error bars indicate the mean ± SD, n = 3. *C*, analysis of H240R-Nb82-mediated blocking of ASFV attachment and internalization to PAM cells using confocal microscopy. *Gray boxed* regions indicate representative cells selected for magnified views (*blue*, nuclei; *green*, viral particles; *red*, β-actin.) Scale bars: 50 μm in low-magnification images and 10 μm in magnified images. *D*, fluorescence intensity quantification of ASFV attachment and internalization was performed by IFA and confocal microscopy using ImageJ. The bar chart shows the ASFV-positive signal intensities. Three randomly selected fields of view per group were analyzed in each independent experiment, and three independent experiments were performed. Data are presented as mean ± SD. ∗∗∗∗*p* < 0.0001. *E*, HAD assay analysis of H240R-Nb82 inhibition of progeny ASFV production after the attachment stage. The p72-Nb2 served as an irrelevant nanobody control. Viral titers were determined at 48 h postattachment assay and expressed as logHADU_50_/ml. The data shown are representative of three replicate wells (two-tailed paired Student’s *t* test). Error bars indicate the mean ± SD, n = 3. ASFV, African swine fever virus; HAD, hemadsorption; HADU50/ml, 50% hemadsorption unit per mL; IFA, immunofluorescence assay; ns, not significant; PAMs, porcine alveolar macrophages.

To elucidate the neutralizing mechanism of H240R-Nb82, we assessed its impact on early ASFV infection using the viral attachment and internalization assays. The qPCR results showed that H240R-Nb82 significantly reduced viral attachment compared with the irrelevant nanobody p72-Nb2 (Fig. 5*A*). However, the viral internalization did not show a significant change (Fig. 5*B*). Confocal microscopy was further performed. Quantitative analysis of fluorescence signals showed that treatment with 60 μg H240R-Nb82 reduced peripheral viral signals, with no significant reduction in internalization, indicating inhibition of viral attachment (Fig. 5, *C* and *D*). Consistently, 50% hemadsorption dose (HADU_50_) assay showed that H240R-Nb82 reduced progeny virus titers by approximately one logHADU_50_/ml compared with the ASFV-only and p72-Nb2 control groups, whereas p72-Nb2 had no significant effect (Fig. 5*E*). These results indicate that the H240R-Nb82 interferes with the attachment stage of ASFV infection, inhibiting the virus's binding to host cells, thereby limiting ASFV replication.

### Identification of the neutralizing epitope of ASFV H240R

**Figure 6:**
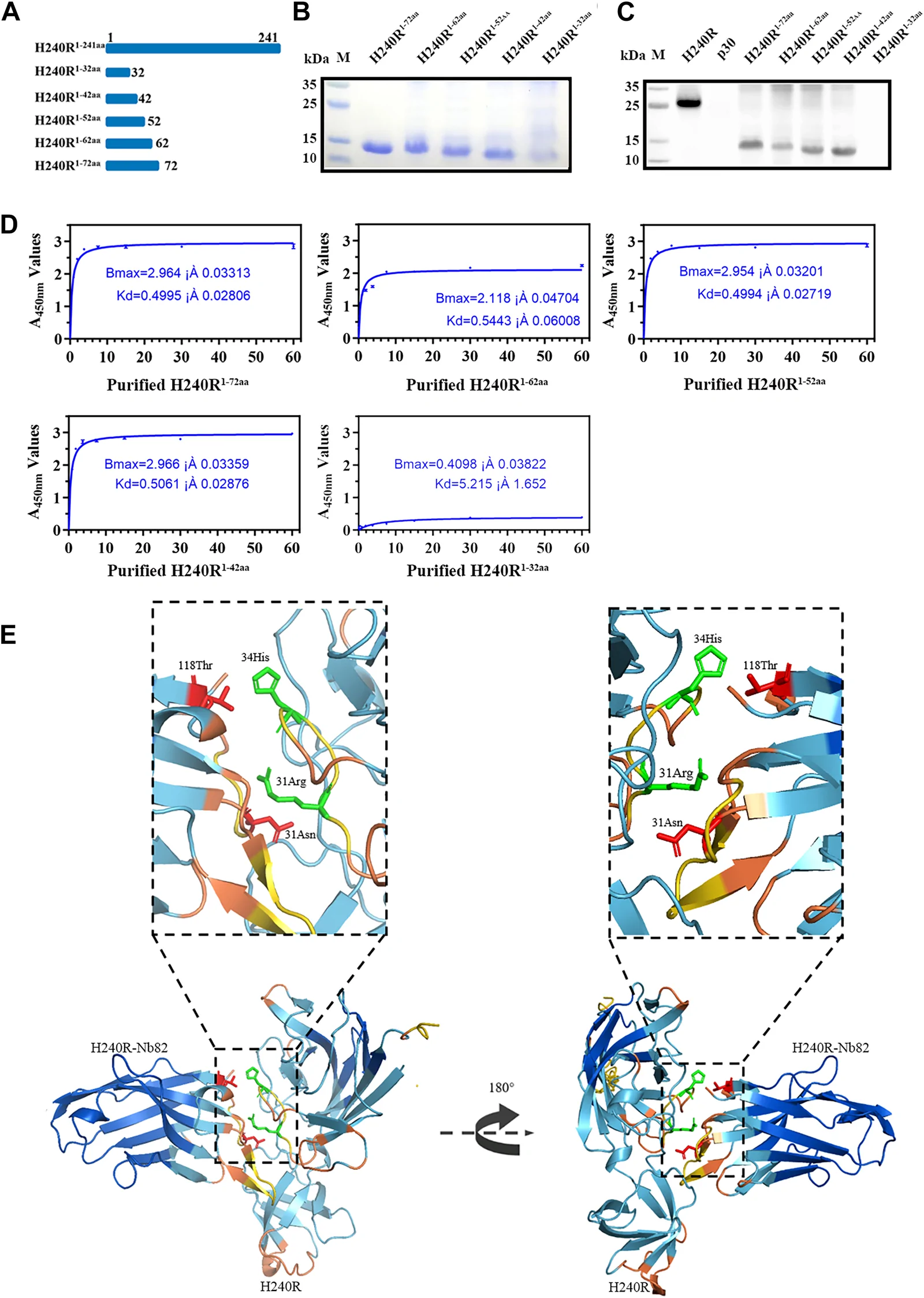
**Fine epitope mapping of the neutralizing epitope recognized by the H240R-Nb82.***A*, schematic of the different truncation constructs of ASFV H240R. *B*, SDS-PAGE analysis of the expression and purification of the different truncated H240R proteins in *E. coli* BL21 (DE3) and purified by Ni-NTA affinity chromatography. *C*, Western blotting analysis of H240R-Nb82-HRP fusion protein binding to different truncated H240Rs. *D*, ELISA-based binding analysis of H240R-Nb82-HRP to the different truncated H240R proteins. Bmax values and Kd values were calculated by nonlinear regression. Error bars indicate the mean ± SD, n = 3. *E*, computational structural model of the H240R-Nb82 complex generated using AlphaFold3 and visualized in PyMOL. Two views related by a 180° rotation are shown. H240R is colored according to the pLDDT confidence scores. H240R residues Arg31 and His34 are highlighted in *green*, whereas H240R-Nb82 residues Asn31 and Thr118 are highlighted in *red*. *Dashed boxes* show enlarged views of the putative binding interface. The model is presented as a qualitative structural reference rather than an experimentally validated structure. HRP, horseradish peroxidase; pLDDT, predicted local distance difference test.

Given the competitive binding and neutralizing activity of H240R-Nb82, we identified its target epitope. For preliminary mapping, H240R was experimentally divided into three larger fragments (H240R^1-90aa^, H240R^73-168aa^, and H240R^151-241aa^), which were expressed in *E. coli* BL21 (DE3), and their binding activities to H240R-Nb82 were evaluated by ELISA. The N-terminal fragment showed the obvious binding signal, indicating that the Nb82-binding region was located within the N-terminal one-third of H240R (Fig. S4). Based on this result, a series of C-terminal truncations of the H240R protein named H240R^1-72aa^, H240R^1-62aa^, H240R^1-52aa^, H240R^1-42aa^, and H240R^1-32aa^ were designed and expressed in *E. coli* BL21 (DE3) (Fig. 6*A*). SDS-PAGE confirmed expression of all constructs, although the shortest preparation showed lower purity than the others (Fig. 6*B*). SEC was performed to assess the solution-state homogeneity and aggregation status of the purified full-length and truncated H240R proteins. Most protein preparations showed predominant main elution peaks with only minor early-eluting species, indicating good solubility and limited aggregation (Fig. S5). However, the H240R^1-32aa^ preparation displayed multiple bands on SDS-PAGE (Fig. 6*B*), suggesting that its purity was lower than that of the other constructs. Using the H240R-Nb82-HRP fusion protein as the detection antibody, Western blotting analysis showed that H240R-Nb82 bound to four truncations H240R^1-72aa^, H240R^1-62aa^, H240R^1-52aa^, and H240R^1-42aa^ but not to H240R^1-32aa^ (Fig. 6*C*). ELISA-based quantification yielded Bmax (*A*_450nm_) values of 2.96 ± 0.03 (H240R^1-72aa^), 2.12 ± 0.05 (H240R^1-62aa^), 2.95 ± 0.03 (H240R^1-52aa^), 2.97 ± 0.02 (H240R^1-42aa^), and 0.41 ± 0.04 (H240R^1-32aa^) for the five respective constructs, confirming a marked loss of binding to the shortest truncation H240R^1-32aa^ (Fig. 6*D*). These results indicated that the epitope recognized by the H240R-Nb82 located at residues 33 to 42, with the sequence ^33^GHYPFSFELK^42^. To further determine whether the Nb82-recognized region could be recapitulated as a linear epitope, a synthetic H240R^25-45aa^ peptide spanning the mapped N-terminal region was prepared and examined for binding to H240R-Nb82. Dot blot analysis showed that H240R-Nb82 specifically recognized both the synthetic H240R^25-45aa^ peptide and the full-length H240R protein, whereas no detectable binding was observed with the irrelevant control or the negative control (Fig. S6). These results demonstrate that the H240R-Nb82 binds to a linear peptide sequence, further supporting the identification of a linear epitope within the N-terminal region of H240R. To complement the experimental epitope-mapping results, we performed a computational structural prediction of the H240R–Nb82 complex using AlphaFold3 without applying any predefined epitope restraints or interface constraints (Fig. 6*E*). We emphasize that this prediction is entirely computational and is presented only as a qualitative visualization aid rather than definitive structural evidence. The model tentatively suggests that Arg31 and His34 of H240R may be positioned near Asn31 in CDR1 and Thr118 in CDR3 of H240R-Nb82, respectively.

**Figure 7:**
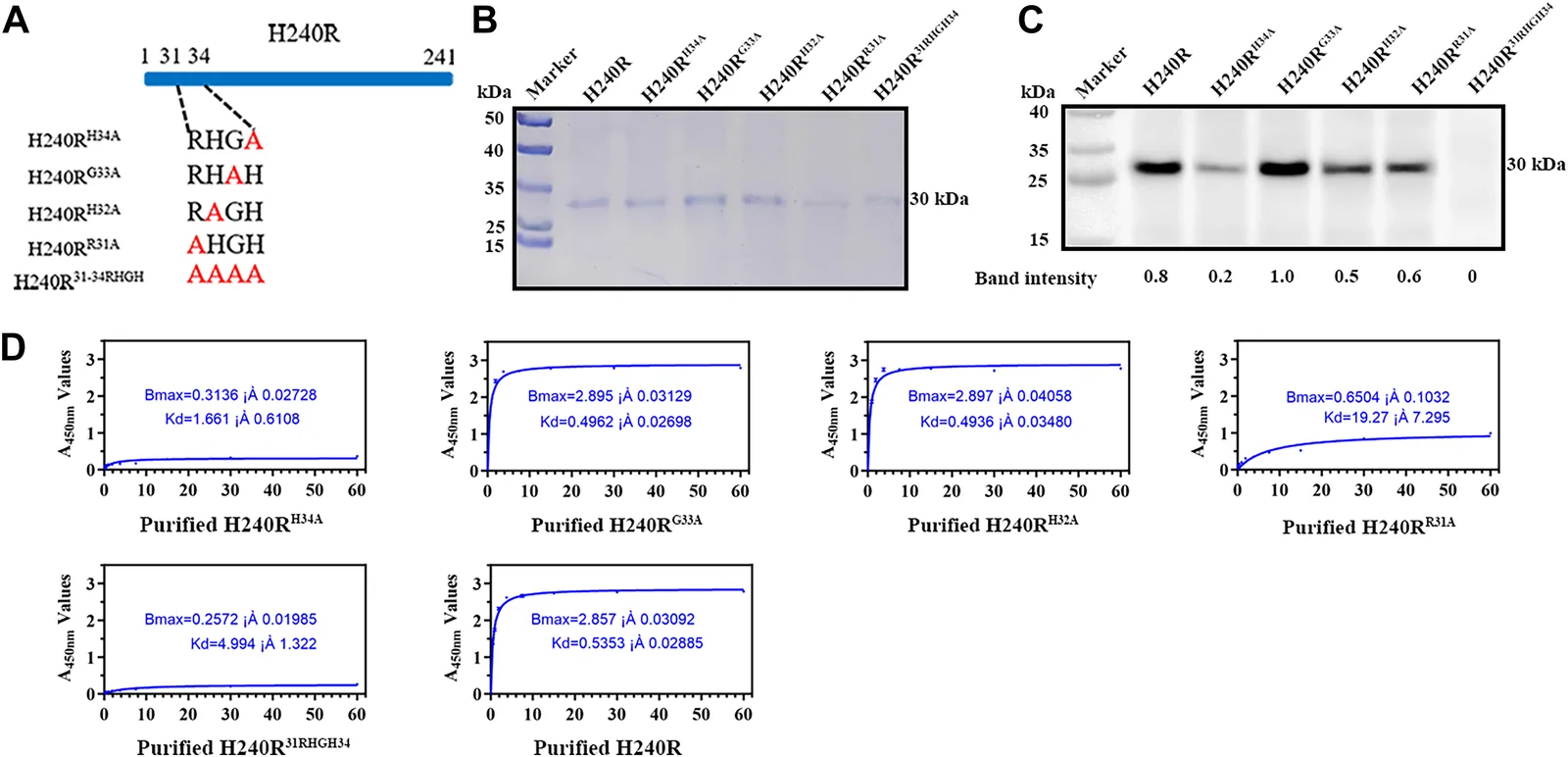
**His34 is an important residue for H240R-Nb82 binding.***A*, schematic diagram of ASFV H240R mutants carrying single or combined substitutions at residues 31 to 34. *B*, SDS-PAGE analysis of recombinant H240R mutants expressed in *E. coli* BL21 (DE3) and purified by Ni-NTA affinity chromatography. *C*, Western blotting analysis of the H240R-Nb82 binding to different mutated H240Rs. *D*, ELISA analysis of the maximum binding ability between each mutated H240R and the H240R-Nb82. Bmax values and Kd values were calculated by nonlinear regression. Error bars indicate the mean ± SD, n = 3. ASFV, African swine fever virus.

To experimentally assess the contribution of these residues to H240R-Nb82 binding, we introduced point mutations (R31A, H32A, G33A, and H34A) and a combined mutation (R31A/H32A/G33A/H34A) within the epitope region, followed by expression and purification of the corresponding H240R mutant proteins (Fig. 7, *A* and *B*). Western blotting with single-point and combination mutations as antigens revealed that while the R31A mutation weakened nanobody binding, the H34A mutation had a more pronounced effect (Fig. 7*C*). When all residues were mutated simultaneously, binding capacity was completely abolished (Fig. 7*C*). To further validate the contribution of these residues at the peptide level, synthetic H240R^25-45aa^ peptides carrying the corresponding single or combined substitutions were prepared, and dot-blot analysis showed a binding pattern consistent with that observed for the full-length mutant proteins (Fig. S7). Consistent with this, ELISA measurements demonstrated that the Bmax values of the H34A mutant and the combined mutation were markedly reduced (0.26 ± 0.020 and 0.31 ± 0.027), while the H32A and G33A mutants and the unmutated H240R maintained relatively high levels (2.90 ± 0.0401, 2.90 ± 0.0312, and 2.86 ± 0.0309) (Fig. 7*D*). These results suggest that His34 is an important residue for H240R-Nb82 recognition and binding to H240R and that substitution at this position has a pronounced effect on the interaction. Moreover, our direct ELISA using ASFV particle-coated plates and capture ELISA showed that H240R-Nb82 can bind purified virions under the experimental conditions used in this study. To further evaluate the conservation of this region across different ASFV genotypes, we performed sequence alignment of the H240R protein from representative ASFV isolates. The results showed that the displayed residues, including the nanobody-recognized site, are conserved among the analyzed strains, supporting the conservation of this region across these ASFV genotypes (Fig. S8). Collectively, these results indicate that H240R-Nb82 recognizes a conserved linear epitope on H240R (residues ^33^GHYPFSFELK^42^).

### Immunogenicity of H240R epitope analysis

**Figure 8:**
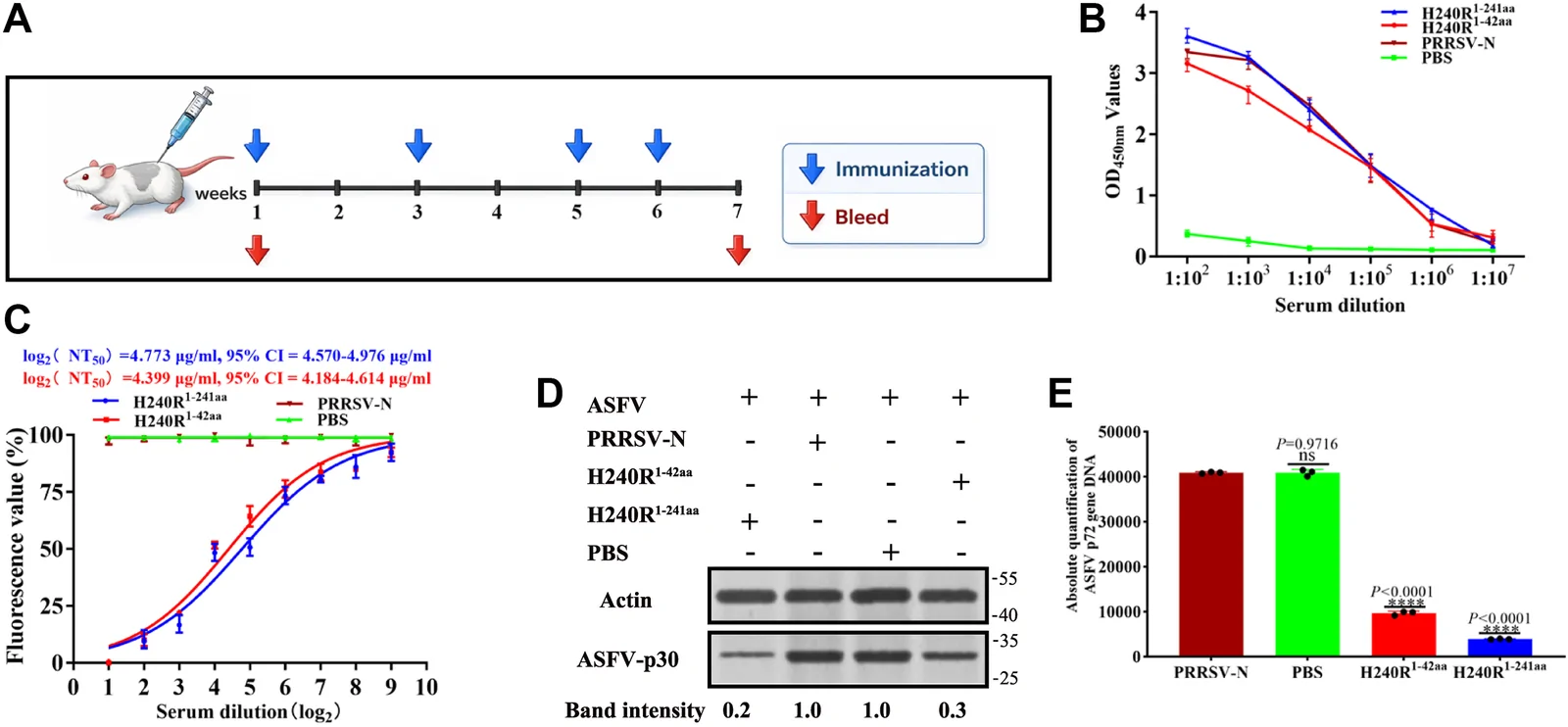
**The neutralizing epitope in H240R recognized by the H240R-Nb82 elicits neutralizing antibody responses in mice.***A*, schematic diagram of the mouse immunization schedule with H240R proteins (H240R^1-241aa^ and H240R^1-42aa^); PRRSV-N and PBS were used as irrelevant antigen and negative control, respectively. *B*, serum antigen-specific IgG titers against the corresponding immunogens determined by indirect ELISA. Error bars indicate the mean ± SD, n = 6. *C*, virus neutralization assay and NT_50_ determination by IFA. Fluorescence values at serial 2-fold serum dilutions (log_2_ scale) were measured, and NT_50_ values were calculated from curve fitting of the fluorescence-dilution curves (GraphPad Prism). Error bars indicate the mean ± SD, n = 3. *D*, Western blotting analysis of the neutralizing effect of mouse sera against CN/GS/2018 strain. ASFV-infected cells were analyzed for ASFV-p30 levels after the virus was preincubated with sera from different groups. *E*, qPCR analysis of the neutralizing effect of mouse sera against CN/GS/2018 strain. Absolute quantification of ASFV p72 DNA was performed on nucleic acids extracted from ASFV-infected cells treated with sera from each group. The data shown are representative of three replicates (ordinary one-way ANOVA). Error bars indicate the mean ± SD, n = 3. ∗∗∗∗*p* < 0.0001. ASFV, African swine fever virus; IFA, immunofluorescence assay; ns, not significant; NT50, 50% neutralization titer; pLDDT, predicted local distance difference test; PRRSV, porcine reproductive and respiratory.

To further assess the immunogenic and functional relevance of the Nb82-mapped H240R epitope-containing region in an independent *in vivo* model, BALB/c mice were divided into four groups, including the H240R^1-42aa^ truncated H240R proteins immunization group, the H240R^1-241aa^ full-length protein immunization group, the porcine reproductive and respiratory syndrome virus (PRRSV)-N protein antigen control group, and the PBS group (Fig. 8*A*). Serum samples were collected in the seventh week for subsequent analysis.

Serum-specific antibody titers were measured by ELISA, and the results showed that both the H240R^1-42aa^ and H240R^1-241aa^ immunization groups had higher antibody levels compared to the PBS-negative control group, and the PRRSV-N protein irrelevant antigen control group also exhibited higher antibody levels (Fig. 8*B*). Notably, the H240R^1-241aa^ group exhibited higher *A*_450nm_ values at lower serum dilutions, suggesting a stronger immunogenicity (Fig. 8*B*). A fluorescence-based neutralization assay was performed to assess the neutralizing activity of the serum samples. The results showed that the sera from the H240R^1-42aa^ and H240R^1-241aa^ immunized groups retained potent neutralizing activity at higher serum dilutions (Fig. 8*C*). However, no neutralizing activity was detected in the PBS or PRRSV-N control groups (Fig. 8*C*). Specifically, the 50% neutralization titers for the H240R^1-42aa^ and H240R^1-241aa^ groups were 2^4.773^ and 2^4.399^, respectively (Fig. 8*C*). Western blotting analysis further confirmed that the immune sera from both the H240R^1-42aa^ and H240R^1-241aa^ groups effectively neutralized ASFV and significantly inhibited the expression of p30 protein (Fig. 8*D*). The qPCR quantification also showed that the ASFV p72 DNA copy numbers in cells treated with sera from both groups were significantly lower than those from the PBS control and PRRSV-N groups (Fig. 8*E*). These findings demonstrate that the truncated H240R^1-42aa^ protein containing the identified epitope is capable of inducing functional antibodies that inhibit viral replication, with the full-length protein context yielding a comparable immune response. In summary, this conserved epitope possesses both immunogenicity and functional activity.

## Discussion

Neutralizing epitopes are critical targets for vaccine and antiviral development (20). However, their precise identification in ASFV has remained challenging (21). Although several major structural proteins, including p30, p54, and p72, have been extensively studied and can provide partial protection in pigs with these proteins expressed *in vitro*, functionally validated neutralizing epitopes remain limited (22). In this study, using a reverse vaccinology-guided strategy integrating nanobody screening (23), functional characterization, and epitope mapping, we identified a neutralizing nanobody targeting the ASFV capsid protein H240R and defined a previously unrecognized conserved neutralizing epitope. Our indirect ELISA using ASFV particle-coated plates and capture ELISA showed that H240R-Nb82 directly binds purified virions under the experimental conditions used in this study.

ASFV H240R has previously been described as a structural protein involved in ASFV virion assembly (24). However, the function of H240R has rarely been reported. Here, we identified a nanobody targeting H240R protein, H240R-Nb82, that exhibits robust neutralizing activity against different genotype of ASFV isolates. This finding expands the repertoire of known ASFV proteins to harbor functional neutralizing epitopes and suggests that H240R can serve as a relevant target for developing ASFV subunit vaccines. Nevertheless, because the current evidence is derived primarily from *in vitro* assays, further *in vivo* studies in pigs are required to validate its protective efficacy.

Systematic truncation and alanine-scanning mutagenesis showed that the neutralizing epitope recognized by H240R-Nb82 is a linear region spanning residues ^33^GHYPFSFELK^42^, with His34 being critical for binding. Antibody–antigen binding is generally mediated by multiple interactions across an epitope, and His34 is more likely to represent an important residue within the broader ^33^GHYPFSFELK^42^ linear epitope. The reason why His34 plays a relatively important role in this epitope remains to be further clarified, and future high-resolution structural studies and more detailed mutagenesis analyses will be required to define its precise contribution to H240R-Nb82 recognition. Notably, these sequences of the epitope were fully conserved among 38 ASFV strains from diverse genetic lineages. Consistent with this conservation, H240R-Nb82 neutralized three genetically distinct ASFV isolates, whereas another nanobody, H240R-Nb13, displayed genotype-restricted activity. This is likely due to variations in the specific epitope it recognizes within the H240R protein across different ASFV genotypes. Future research should include a broader range of viral strains to validate the effectiveness of this epitope against more diverse isolates, as the number of strains tested in this study remains limited.

Mechanistically, our data were most consistent with H240R-Nb82 acting at an early pre-entry stage, likely during attachment. ASFV possesses a complex, multilayered architecture, including an outer envelope, a capsid, and an inner membrane (12). The results of indirect ELISA with the coating ASFV particles, capture ELISA, and structural prediction of the binding interaction between the nanobody and H240R in this study all confirm that ASFV particles can exist with an exposed capsid and that H240R is located on the outer capsid surface. Notably, the ASFV particles used in this study were purified by sucrose density gradient centrifugation, a method previously reported to potentially disrupt the viral outer envelope, thereby exposing the capsid and allowing direct binding of the nanobody to purified virions (12). Previous studies have shown that viral particles can remain infectious even in the absence of the outer envelope (25). Capsid-exposed infectious ASFV particles may originate from an intracellular infectious form lacking the outer membrane or from extracellular virions whose outer membrane is disrupted during entry (10). We have confirmed that H240R-Nb82 interacts with the capsid protein H240R, blocks the viral attachment process, and reduces progeny virus production. This result further demonstrates that ASFV, even after losing its outer envelope and retaining only the capsid, remains capable of infecting host cells. However, direct molecular evidence is still lacking to determine whether this effect is mediated through competitive inhibition of interactions between H240R and host factors or through alterations in virion surface conformation. Moreover, the spatial context of the identified linear epitope within the native conformation of H240R, as well as its accessibility on intact virions, may critically influence neutralization efficiency and immunogenicity. Future studies combining kinetic approaches, such as surface plasmon resonance (26), with high-resolution structural methods, including cryo-electron microscopy or X-ray crystallography, will be required to resolve the complex of H240R-Nb82 and H240R and elucidate the neutralization mechanism from a structural perspective.

Although ASFV evolves slowly, there are still differences between genotypes (27). As we know, the live-attenuated vaccine is a promising strategy against ASFV. However, its efficacy against emerging variant strains, especially naturally recombinant ones, is not guaranteed. Challenge experiments have demonstrated that gene-deleted vaccines derived from genotype II parental strains (*e.g.*, those lacking EP402R) cannot fully protect animals from genotype Ⅰ, Ⅱ recombinant strains (28). This indicates that genetic recombination may alter the virus's key antigenic epitopes. Here, the H240R-Nb82, which targets a conserved epitope, could effectively neutralize genotype Ⅰ, genotype Ⅱ, their recombinant isolates, and deleted ASFV isolates. This result confirms that the targeted epitope is highly conserved and functionally important throughout viral evolution. Therefore, H240R-Nb82 serves as both a vital research tool and a foundation for developing broad-spectrum anti-ASFV antibodies. However, a limitation of this study is that only a single isolate from each genotype was selected, and the number of viral strains tested was limited, which may not fully represent the diversity of ASFV strains. Future research should include a broader range of viral strains to validate the effectiveness of this epitope against more diverse isolates.

Sequence analysis of the H240R-specific nanobodies showed that four out of five sequences contained an additional cysteine residue within CDR3, and several sequences also contained cysteine residues within CDR1. The conserved cysteine near the FR3-CDR3 boundary is expected to participate in the canonical intradomain disulfide bond and was therefore not considered a candidate for additional pairing (29). Instead, the presence of a noncanonical cysteine in CDR1 together with another located further within CDR3 is consistent with the possible formation of a camelid VHH-associated H1-H3 disulfide bond, a feature that is generally absent from conventional IgG antibodies and may contribute to stabilization of the single-domain antibody fold in the absence of a light chain (30). Among these H240R-specific nanobodies, H240R-Nb35, H240R-Nb55, and H240R-Nb82 contain cysteine residues in both CDR1 and the internal region of CDR3 (cysteine framed in red), suggesting that it may possess this characteristic VHH-associated disulfide bond; however, its formation and connectivity have not been experimentally confirmed. The ability of H240R-Nb82 to recognize the identified epitope may be related to the structural characteristics of nanobodies. Compared with traditional monoclonal antibodies, nanobodies are smaller and structurally simpler, allowing them to be more flexible in antigen recognition and able to access concealed epitopes on the viral surface that traditional antibodies cannot reach (31). Additionally, nanobodies exhibit excellent stability and solubility and are easy to engineer and express on a large scale, providing convenience for practical applications (32). The H240R-Nb82 could also be further optimized in the future by fusing with pig IgG Fc fragments to extend its half-life or designing bispecific antibodies to enhance neutralization efficiency and expand their protective range (33). Overall, this study provides a new candidate molecule for the prevention and treatment of ASFV, while also reinforcing the great potential of nanobodies in discovering viral vulnerabilities and developing novel biopharmaceuticals.

## Experimental procedures

### Cells, vectors, and viruses

HEK293T cells were cultured in Dulbecco’s Modified Eagle Medium (Thermo Fisher Scientific) containing 10% fetal bovine serum (FBS, Gibco) and 1% penicillin-streptomycin, at 37 °C under a humidified atmosphere of 5% CO_2_. PAMs were isolated from 6-week-old pigs by bronchoalveolar lavage following an established protocol (34) and maintained in RPMI 1640 medium (Biological Industries, Sartorius AG) supplemented with 10% FBS, under the same culture conditions (37 °C, 5% CO_2_, humidified atmosphere).

The pMECS vector, a gift from Professor Serge Muyldermans, was used for VHH library construction according to established methods (35). To produce nanobody-HRP fusion proteins, the enhanced green fluorescent protein cassette in the pCMV-N1-enhanced green fluorescent protein plasmid was replaced with a HRP-coding sequence, resulting in the pCMV-N1-HRP construct (36).

The ASFV strains were used in the study including virulent CN/GS/2018 strain (genotype II), 1063 strain (genotype I), 731 strain (genotype I/II recombinant strain), and a two-gene-deleted 2ΔASFV strain (genotype II). All work involving ASFV was conducted in the institute’s biosafety level 3 laboratory of the Lanzhou Veterinary Research Institute, Chinese Academy of Agricultural Sciences.

### Cloning, expression, and purification of recombinant ASFV H240R proteins and its variants

The gene encoding ASFV H240R (GenBank accession no. XQL01838.1) was commercially synthesized by GenScript and cloned into the pUC56 vector, yielding the construct pUC56-H240R. The H240R coding sequence was then amplified by PCR with the primers ASFV-H240R-F and ASFV-H240R-R (Table S1) and inserted into the pET-21b(+) vector (Novagen) using the *BamH*I and *Xho*I restriction sites (New England Biolabs).

After verifying the plasmid sequence, it was transformed into *E. coli* (*E. coli*) BL21(DE3) cells (TransGen Biotech) for expression. Bacterial cultures were grown to an *A*_600nm_ of 0.6 to 0.8, and expression was induced with 0.5 mM IPTG at 37 °C for 6 to 8 h. Cells were harvested by centrifugation, resuspended in PBS, and lysed by sonication on ice (35 W, 20 kHz, 3 s pulses interspersed with 3 s pauses for a total of 50 min).

The lysate was centrifuged at 6000×*g* for 15 min, and the insoluble fraction was solubilized in denaturing buffer (8 M urea, 100 mM NaH_2_PO_4_, 100 mM Tris·HCl, pH 8.0). The His-tagged recombinant H240R protein was purified under denaturing conditions using Ni-NTA affinity chromatography (Smart-Lifesciences) according to the manufacturer’s instructions. Subsequently, the denatured protein was refolded by stepwise dialysis against buffers with progressively decreasing urea concentrations and finally dialyzed into PBS. Protein concentration, purity, and antigenicity were analyzed by ELISA, SDS-PAGE, and Western blotting.

The genes encoding C-terminally truncated variants and point mutants of H240R were generated by PCR amplification using specific primers (Table S1). Truncated genes (H240R^1-72aa^, H240R^1-62aa^, H240R^1-52aa^, H240R^1-42aa^, and H240R^1-32aa^) were cloned into pET-21b(+) with a C-terminal His tag. Site-directed mutagenesis was performed to introduce R31A, H32A, G33A, H34A, and the combined mutant (R31A/H32A/G33A/H34A), and all constructs were verified by sequencing. For proteins expression, the recombinant bacteria were cultured to mid-log phase (*A*_600nm_ = 0.6–0.8), and expression was induced with IPTG (0.1 mM) for 8 h. The induced bacterial cells were harvested by centrifugation, resuspended in prechilled lysis buffer, and placed in an ice-water mixture for sonication. Lysates were centrifuged at 4 °C to separate soluble and insoluble fractions. The target proteins were predominantly present in the insoluble pellet as inclusion bodies. Inclusion bodies were collected by centrifugation (15,000 × *g*, 30 min) and solubilized in denaturing buffer containing 8 M urea. After clarification by centrifugation, these His-tagged proteins were purified under denaturing conditions using Ni-NTA affinity chromatography, washed with imidazole-containing wash buffer, and eluted with high-imidazole elution buffer.

The homogeneity and aggregation status of the purified full-length and truncated H240R proteins were further analyzed by SEC. Briefly, purified protein samples were equilibrated in PBS, pH 7.4, and filtered through a 0.45 μm membrane before injection. SEC was performed on a Shimadzu LC-16 system equipped with a UV detector and a gel filtration chromatography column. The mobile phase consisted of PBS, pH 7.4, and separation was carried out under isocratic elution at a flow rate of 1.0 ml/min. The elution profiles were monitored at 280 nm.

### Camel immunization and nanobody library construction

An adult male Bactrian camel (4-year-old) was immunized with five rounds of purified recombinant ASFV H240R protein according to the established methods (37). Before immunization, the endotoxin level of the final recombinant H240R protein preparation was measured and confirmed to be 4 EU/mg of protein, which met the acceptable standard for animal immunization. The initial immunization used 1 mg of protein (2 mg/ml) emulsified in Freund’s complete adjuvant. Following, four booster injections were performed and each administered at 2-week intervals using the same amount of antigen in Freund’s incomplete adjuvant. Serum samples were collected periodically to monitor anti-H240R antibody titers by indirect ELISA as previously described (19). Five days after the final immunization, 200 ml of peripheral blood was drawn, and lymphocytes were isolated using Leucosep tubes (Greiner Bio-One) according to the manufacturer’s instructions.

Total RNA was extracted from approximately 3 × 10^7^ lymphocytes using the RNeasy Plus Mini Kit (QIAGEN GmbH). First-strand cDNA was synthesized with the SuperScript III First-Strand Synthesis System (Invitrogen). The VHH gene fragments were amplified *via* nested PCR which was an initial round with primers CALL001 and CALL002 (Table S1) yielded a 700 bp product, and then re-amplified using VHH-F and VHH-R primers (Table S1) to generate approximately 400 bp VHH genes.

The purified VHH fragments were digested with *Pst*I and *Not*I (New England BioLabs) and ligated into the similarly digested phagemid vector pMECS using T4 DNA ligase. The ligated product was electroporated into *E. coli* TG1 cells. Transformed cells were plated on Luria-Bertani (LB) agar containing 2% glucose and 100 μg/ml ampicillin and incubated at 37 °C for 6 to 8 h. Colonies were harvested and stored at −80 °C in 20% (v/v) glycerol. Library size was determined by plating a diluted aliquot of the transformed cells and counting colony-forming units. To assess library quality, 48 randomly selected clones were screened by PCR using primers VHH-F and VHH-R to estimate the insertion efficiency.

### Screening of H240R-specific nanobodies

The phage display library was subjected to biopanning nanobodies targeting the ASFV H240R protein, following previously described methods with minor modifications (35). The library was first rescued by infecting *E. coli* TG1 cells with M13K07 helper phage at a multiplicity of infection (MOI) of 20. For each round of biopanning, the ELISA plates were coated with purified H240R protein (10 μg/well; four wells per round) and incubated overnight at 4 °C. After washing three times with PBST (PBS with 0.05% Tween-20), the wells were blocked with 200 μl of blocking buffer (5% skim milk in PBST) for 1 h at 37 °C. Following another series of washes, the rescued phage library was added and incubated for 2 h at 25 °C. Bound phages were eluted with 100 μl of 100 mM trimethylamine for 10 min at room temperature and immediately neutralized with 100 μl of 1 M Tris-HCl (pH 7.4). The eluted phage particles were used to infect log-phase *E. coli* TG1 cells for subsequent screening.

After three rounds of biopanning, enrichment for H240R-specific phages was monitored by phage titer measurement and ELISA. Ninety-six individual colonies from the third round were randomly selected for production of crude periplasm extracts containing nanobodies. Briefly, each clone was initially grown in 100 μl of LB medium supplemented with ampicillin and glucose (LB/AMP-GLU) in a 96-well plate at 37 °C for 6 h. Then, 10 ul of the culture was transferred to 24-well plates containing 1 ml of terrific broth medium per well and grown until *A*_600nm_ reached 0.6 to 0.8. Protein expression was induced by 1 mM IPTG overnight at 37 °C. Periplasmic extracts containing soluble nanobodies were obtained by freeze-thaw cycling (three repetitions) followed by centrifugation. The binding specificity of each extract was evaluated by indirect ELISA using H240R as the coating antigen, and p30 (CP204L) protein was used as coating antigen for negative control. Clones showing strong H240R-specific binding were selected for sequencing. The VHH sequences obtained by sequencing were annotated according to the IMGT numbering scheme for antibody variable domains, and the CDR1, CDR2, and CDR3 boundaries were assigned based on the IMGT definition.

### Expression and characterization of nanobody-HRP fusion proteins against ASFV-H240R protein

The nanobody-HRP fusion proteins were expressed as described previously (38). Briefly, the positive recombinant pMECS vectors carrying VHH genes and the pCMV-N1-HRP vector (containing HA and His tags) were codigested with *Pst*I and *Not*I restriction enzymes (New England BioLabs). The VHH fragments were then ligated into the linearized pCMV-N1-HRP backbone. After sequencing, the positive plasmids were transfected into HEK293T cells using polyethyleneimine agents (Polysciences Inc.). At 48 h post-transfection, expression of the fusion proteins was assessed by IFA. Culture supernatants were collected and filtered through 0.22 μm cellulose acetate membranes. The specific reaction between the nanobody-HRP in the culture supernatants, and H240R protein was evaluated by direct ELISA.

### Prokaryotic expression of nanobodies against ASFV H240R

The VHH genes encoding nanobodies were subcloned into the pET-28a(+) vector and transformed into *E. coli* BL21(DE3) competent cells. The expression of the recombinant nanobodies was performed following the same procedure as described for the truncated H240R variants. For nanobody preparations, eluted proteins were refolded by stepwise dialysis against refolding buffers with gradually decreasing denaturant concentrations, followed by a final buffer exchange into storage buffer (*e.g.*, PBS, pH 7.4). Residual insoluble aggregates were removed by centrifugation. The purified proteins were stored at −80 °C for subsequent experiments.

### Proliferation and purification of ASFV particles

Primary PAMs were infected with the ASFV CN/GS/2018 strain at a MOI of 1 and incubated at 37 °C for 5 days. Then, the cells were subjected to three freeze-thaw cycles to release the virus, followed by centrifugation at 3000×*g* for 10 min to remove large cellular debris. The supernatant was transferred to an ultracentrifuge tube and centrifuged at 150,000×*g* for 4 h to collect the viral pellet, and the pellet was resuspended in 1.5 ml PBS. A sucrose gradient was established by layering 2 ml of 50%, 40%, 30%, and 20% sucrose solutions, followed by 1.5 ml of the resuspended sample. The virus was purified by ultracentrifugation at 150,000×*g* for 4 h. After ultracentrifugation, the virion-containing region corresponding to the 25% to 56% sucrose portion of the gradient was collected. The virus was resuspended in PBS and further purified by centrifugation at 100,000×*g* for 2.5 h to remove sucrose, followed by resuspension in PBS. The purified ASFV was stored at −80 °C.

### ELISA analysis

To determine antibody titers, serum samples from the immunized camel, as well as periplasmic extracts and culture supernatants, were analyzed by indirect ELISA as described previously (39). Briefly, the ELISA plates were coated with 400 ng per well of purified different H240R proteins in PBS and incubated overnight at 4 °C. After blocking and washing, serial dilutions of serum, periplasmic extracts, or supernatants were added and incubated for 1 h at 37 °C. For serum samples, plates were subsequently incubated with rabbit anti-camel IgG antibodies (1:5000 dilution), followed by HRP-conjugated goat anti-rabbit IgG (1:10,000; Beijing TransGen Biotech). Periplasmic extracts were detected using an anti-HA monoclonal antibody (1:2000) and HRP-conjugated goat anti-mouse IgG (1:5000; Beijing TransGen Biotech).

To analyze the nanobodies activity and identify the binding epitopes, ELISA was performed. Wells were coated with purified complete, truncated, and mutated ASFV H240R proteins. Binding was detected using the intrinsic peroxidase activity of the fusion protein. Supernatant containing nanobody-HRP fusions required no secondary antibody. After extensive washing with PBST, color development was initiated with TMB substrate for 15 min and stopped with 3 M H_2_SO_4_. Absorbance was measured at 450 nm (*A*_450nm_) using a Bio-Rad microplate reader.

To analyze binding of nanobodies to the native conformation of H240R, ELISA was performed. Wells were coated with virus particles. Binding was detected using the intrinsic peroxidase activity of the fusion protein. Supernatant containing nanobody-HRP fusions required no secondary antibody. After extensive washing with PBST, color development was initiated with TMB substrate for 15 min and stopped with 3 M H_2_SO_4_. Absorbance was measured at 450 nm (*A*_450nm_) using a Bio-Rad microplate reader.

For competition assays, the sera collected from ASFV-positive pigs were mixed with various concentrations of nanobodies and added to the ELISA plate coated with purified H240R protein. After incubating for 1 h at 37 °C, the remaining steps followed the same protocol described above. This competitive assay was performed to evaluate whether the epitope bound by the nanobody is immunogenic.

A nanobody-based capture ELISA was established for analysis of binding of nanobodies to ASFV particles. Nanobodies (800 ng/well) were coated into 96-well plates overnight at 4 °C. After blocking, purified ASFV particles were added and incubated at 37 °C for 1 to 2 h. Plates were washed and incubated with an anti-p30 polyclonal antibody, followed by an HRP-conjugated secondary antibody. Signals were developed with TMB substrate, the reaction was stopped with 3 M H_2_SO_4_, and *A*_450nm_ was measured.

### ASFV neutralization assay

Neutralization activity of nanobody was assessed using PAMs seeded in 24-well plates. Anti-ASFV nanobodies (30 μg) were pre-incubated with the viral particles at an MOI of 0.1 for 2 h at 37 °C under 5% CO_2_. The mixture was then applied to PAMs. After attachment, cells were washed and maintained for 36 h in RPMI-1640 supplemented with 3% FBS. Viral inhibition was evaluated using Western blotting, qPCR, and IFA. To examine dose–response, nanobodies were serially diluted (0–30 μg), mixed with ASFV at an MOI of 0.1, and incubated for 2 h at 37 °C prior to infection. Viral inhibition was evaluated using Western Blotting, qPCR.

### IFA analysis

IFAs were performed to evaluate the expression, antigen-binding capacity of nanobody-HRP fusion proteins, and ASFV neutralization assay, as adapted from previously described methods (39).

HEK293T cells transformed with pCMV-N1-Nbs-HRP were fixed with ice-cold 70% ethanol for 30 min at 4 °C and blocked with 1% BSA for 1 h at 37 °C. After washing with PBS, cells were incubated with an anti-His monoclonal antibody (1:1000; TransGen Biotech) for 1 h at 37 °C, followed by incubation with a FITC-conjugated goat anti-mouse IgG secondary antibody (1:500; Jackson ImmunoResearch) for 30 min. Nuclei were stained with DAPI, and images were acquired using a Leica AF6000 fluorescence microscope.

To assess binding specificity, PAMs were infected with ASFV (GenBank MW393476) at an MOI of 0.1. At 48 h postinfection, the cells were fixed, blocked, and incubated overnight at 4 °C with supernatants containing nanobody-HRP fusion proteins. Bound fusion proteins were detected using the same anti-His and FITC-conjugated secondary antibody and performed according to the protocol described above.

For ASFV neutralization assays, the PAMs were fixed with 4% paraformaldehyde for 30 min at 4 °C and blocked with 1% BSA in PBS for 1 h at 37 °C. After being washed, the cells were incubated with an anti-p30 monoclonal antibody and then with Alexa Fluor 488-conjugated goat anti-mouse IgG (1:500). Nuclei were stained with DAPI. All images were captured using a Leica AF6000 microscope, and each experiment was performed in triplicate.

### Probe-based qPCR

For qPCR, the laboratory-preserved pMD-19T-p72 plasmid was utilized as a positive quantitative standard. Its concentration was measured, and the copy number was calculated according to the formula: Copy number/μl = (N_A_ × concentration [ng/μl] × 10^−9^)/(length of DNA (bp) × 660). The standard was then subjected to 10-fold serial dilutions (10^−1^-10^−9^). qPCR was subsequently performed using the extracted DNA samples as templates, with the specific primers (Table S1) targeting the ASFV-B646L (p72), to enable absolute quantification of viral gene copy numbers in the samples.

### Western blotting

To evaluate nanobody-mediated neutralization of ASFV and dose-dependent inhibition of ASFV, Western blotting analysis was performed. Cell lysates containing equal amounts of protein were separated by SDS-PAGE and transferred onto nitrocellulose membranes. The membranes were blocked for 1 h at room temperature with 5% nonfat milk in PBST, then incubated overnight with a polyclonal anti-p30 antibody. After washing three times with PBST, the membranes were probed for 1 h with HRP-conjugated goat anti-mouse IgG (H + L; 1:5000; Proteintech, SA00001-1). Signals were developed using an ECL Plus kit (Shanghai Genepharma Biotech) and visualized with a Tanon 5200 imaging system. Western blot band intensities were quantified by grayscale densitometric analysis using Image Lab software (Bio-Rad), with the same background-subtraction method applied to all lanes.

### Dot blot assay

To assess the binding of H240R-Nb82 to the synthetic wildtype and mutant peptides, dot blot analysis was performed. The synthetic wildtype H240R^25-45aa^ peptide and the corresponding mutant peptides carrying the R31A, H32A, G33A, H34A, or combined R31A/H32A/G33A/H34A substitutions were synthesized by Tsingke Biotechnology Co., Ltd (China) and spotted onto nitrocellulose membranes. Purified full-length H240R protein was included as a positive control, whereas an irrelevant peptide derived from PRRSV N protein (N^55-75aa^), and PBS were used as negative controls. After air-drying at room temperature, the membranes were blocked with 5% nonfat milk in PBST for 1 h and subsequently incubated with the H240R-Nb82-HRP fusion protein. After three washes with PBST, chemiluminescent signals were developed using an ECL Plus kit.

### Determination of IC_50_

The neutralizing activity of nanobodies against ASFV was quantified using a standardized IC_50_ assay. The ASFV strain (MOI = 0.1) was pre-incubated with 2-fold serial dilutions of nanobody (0–50 μg) for 1 h at 37 °C. The mixture was then applied to PAMs and incubated for 72 h at 37 °C under 5% CO_2_. Viral infection was assessed by IFA targeting the ASFV p30 (CP204L), with four replicate wells per nanobody concentration. Images were analyzed using ImageJ (National Institutes of Health), and the IC_50_ values were calculated using nonlinear regression in GraphPad Prism 10.1.2. All assays were performed in three independent experiments.

### Determination of the CC_50_

The cytotoxicity of H240R-Nb13 and H240R-Nb82 toward PAMs was evaluated using a Cell Counting Kit-8 (CCK-8; Beyotime Biotechnology). PAMs were seeded into 96-well plates and allowed to adhere before treatment with serial dilutions of each nanobody at concentrations of up to 500 μg/ml. Untreated cells served as the cell-viability control, and wells containing culture medium without cells served as blanks.

After incubation for 72 h at 37 °C under 5% CO_2_, CCK-8 reagent was added, and cell viability was determined according to the manufacturer’s instructions. Absorbance was measured at 450 nm using a microplate reader. Nanobody concentrations were log_10_-transformed, and CC_50_ values, defined as the concentrations that reduced cell viability to 50% of that of the untreated control, were calculated by nonlinear regression using GraphPad Prism 10.1.2. Three replicate wells were included for each concentration, and all assays were performed in three independent experiments.

### Prediction of protein–nanobody interactions and epitope mapping

The H240R-Nb82 binary complex was predicted using AlphaFold Server powered by AlphaFold3, using one H240R chain and one Nb82 chain. No predefined epitope restraints, interface constraints, experimentally derived structural restraints, or manual docking procedures were applied during model generation.

Model confidence was assessed using the predicted TM score, interface predicted TM score, predicted aligned error, and predicted local distance difference test (pLDDT) scores. Putative interface residues were located at the predicted interchain contact surface within the predicted model. The model was colored according to the per-atom pLDDT scores provided by AlphaFold3 to visualize prediction confidence. Specifically, the model was colored according to the pLDDT values as follows: blue, pLDDT≥90; light blue, 70<pLDDT <90; yellow, 50<pLDDT <70; and orange, pLDDT<50. Because the atomic coordinates generated from computational prediction do not have sufficient accuracy for quantitative structural interpretation, no atomic distance measurements were extracted or reported. Since this model was generated without experimentally derived structural restraints, it was regarded as a qualitative working model to provide structural context for the experimentally mapped epitope rather than as an experimentally validated structure.

Multiple sequence alignment of ASFV H240R sequences was performed using MegAlign (DNASTAR Lasergene 7.2) to evaluate epitope conservation. Site-directed mutagenesis was performed on selected residues within the experimentally mapped epitope region and/or the putative interface suggested by computational modeling using the primers listed in Table S1. Mutant H240R proteins were expressed and purified as described above. The binding of H240R-Nb82 to the mutant H240R proteins was evaluated by Western blotting and direct ELISA, whereas its binding to the corresponding peptides was assessed by dot blot assay.

### Attachment and internalization assays

Briefly, PAMs were seeded into 24-well plates and cultured for 6 h. The ASFV strain was mixed with nanobodies (120 μg/ml) at a MOI of five and incubated at 37 °C for 1 h, then cooled to 4 °C. The virus-nanobody mixture was added to prechilled PAMs and incubated at 4 °C for 1 h, while control cells were incubated with the virus only. All mixtures were maintained at 4 °C throughout the incubation to prevent internalization. After treatment, the PAMs were washed three times with ice-cold PBS to remove unbound viral particles. Virus attachment to the cell surface was visually detected by IFA. Images were analyzed using ImageJ (National Institutes of Health). Viral DNA levels were quantified by qPCR to determine the number of virions specifically bound to the PAM surface.

The virus internalization assay was performed independently of the virus attachment assay. Briefly, prechilled PAMs were incubated with ASFV alone at a MOI of five for 1 h at 4 °C to allow the virus to attach to the cell surface while preventing viral internalization. The cells were then washed three times with ice-cold PBS to remove unbound viral particles. RPMI 1640 medium containing 3% FBS and nanobody at a final concentration of 120 μg/ml was subsequently added, and the cells were incubated at 37 °C for 2 h to allow viral internalization to proceed in the presence of the nanobody. 50 μg of proteinase K was added into each well and incubated at 4 °C for 45 min. Phenylmethylsulfonyl fluoride was then added to neutralize proteinase K activity, followed by washing with ice-cold PBS. Finally, virus internalization into PAMs was subsequently assessed by IFA. Images were analyzed using ImageJ (National Institutes of Health). Viral DNA copies were quantified by qPCR.

### HAD test

A 1% (v/v) porcine erythrocyte suspension was prepared as follows: blood collected from the cranial vena cava was added into EDTA-containing tube, gently mixed, and centrifuged at 300×*g* for 10 min at 4°C. Plasma and the buffy coat were removed, and the packed erythrocytes were washed three times with four volumes of ice-cold PBS, followed by centrifugation under the same conditions. The washed erythrocytes were resuspended in PBS to a final concentration of 1% (v/v) and stored at 4 °C for use within 24 h.

PAMs were seeded in 96-well plates at a density of 1 × 10^4^ cells per well and allowed to adhere for 6 h. Nanobodies (5 μg/well) and ASFV (MOI = 1) were pre-incubated in 100 μl of RPMI-1640 for 2 h at 37 °C. The mixtures were then added to the PAM monolayers, bringing the total volume to 100 μl per well, and incubated for 2 h. After being washed, 100 μl of maintenance medium (RPMI-1640 containing 3% FBS) was added, and cultures were continued for 48 h. Following this incubation, 20 μl of the 1% porcine erythrocyte suspension was added to each well. Plates were incubated at 37 °C and examined daily for 3 days to observe HAD and cytopathic effects.

To evaluate progeny virus production after the attachment assay, the cells were further incubated at 37 °C for 48 h. Culture supernatants were then collected and subjected to HAD-based endpoint titration on seeded PAMs. Briefly, serial dilutions of the collected supernatants were added to PAMs in replicate wells of 96-well plates. After 36 h of incubation, 20 μl of the 1% porcine erythrocyte suspension was added to each well. Plates were then incubated at 37 °C and examined daily for 3 days for HAD and cytopathic effects. Wells showing clear HAD were scored as positive, and viral titers were expressed as HADU_50_/ml based on endpoint dilution analysis. The procedures for erythrocyte preparation, PAM culture, and HAD readout were performed as described above.

### Immunization of mice and the preparation of serum against full and truncated H240R proteins

In this experiment, BALB/c mice (purchased from Shanxi Huachuang Xinnuo Biotechnology Co., Ltd) were used for immunization. All mice were acclimated for 1 week prior to the study and then randomly assigned to four groups (six mice per group): A PBS negative control group, an H240R^1-42aa^ peptide immunization group, an H240R^1-241aa^ full-length protein immunization group, and a PRRSV-N protein unrelated antigen control group. For immunization, each group was administered the corresponding protein *via* subcutaneous injection in the dorsal region at a dose of 50 μg per mouse, emulsified with Freund's complete or incomplete adjuvant. A total of four immunizations were conducted: the first three were administered at 2-week intervals, followed by a booster immunization in the sixth week. Blood was collected on day 7 after the booster, and serum was separated by centrifugation for both neutralization assays and antibody level detection, performed as previously described.

### Statistical analysis

Data are presented as the mean ± standard deviation (SD). Unless otherwise indicated, n represents the number of independent experiments or biological samples, as specified in the corresponding figure legends; technical replicates within each experiment were averaged to obtain a single value for statistical analysis. Comparisons between two groups were performed using a two-tailed paired Student’s *t* test for matched data or a two-tailed unpaired Welch’s *t* test for independent data, as specified in the corresponding figure legends. Comparisons among three or more matched groups were performed using one-way repeated-measures ANOVA, whereas comparisons among three or more independent groups were performed using ordinary one-way ANOVA. ANOVA was followed by Dunnett’s multiple-comparisons test when multiple groups were compared with a common control. Dose–response curves and IC^50^, CC^50^, or 50% neutralization titer values were determined by four-parameter logistic nonlinear regression. Statistical analyses were performed using GraphPad Prism, version 10.1.2. All tests were two-sided, and *p* < 0.05 was considered statistically significant. Statistical significance is indicated as follows: *p* < 0.05 (∗), *p* < 0.01 (∗∗), *p* < 0.001 (∗∗∗), *p* < 0.0001 (∗∗∗∗), and "ns" denotes no significant difference.

## Ethics statement

Animal experiments were performed based on the Guidance for Experimental Animal Welfare and Ethical Treatment by the Ministry of Science and Technology of China. The protocols of animal experimental procedures were carried out following the guidelines of the Northwest A&F University Institutional Committee for the Care and Use of Laboratory Animals. The procedures were approved by the Committee on Ethical Use of Animals of Northwest A&F University (approval no. 20250023/03).

## Data availability

All data are provided within the article.

## Supporting information

This article contains supporting information.

## Conflict of interest

The authors declare that they have no conflicts of interest with the contents of this article.
