# Supplementary material for: Identification of a conserved neutralizing epitope on the ASFV H240R protein by a nanobody that inhibits viral attachment
Source: J Biol Chem. 2026 Aug 19;302(10):113470. doi: 10.1016/j.jbc.2026.113470 (PMC13590096; doi:10.1016/j.jbc.2026.113470)
Supplement: Supplementary file 1 [file mmc1.pdf]

Identification of a conserved neutralizing epitope on the ASFV H240R protein by a nanobody that inhibits viral attachment

Dian Jiao[1,2,3,4,‡], Binbin Ren[1,2,3,4,‡], Dongying Liu[1,2,3,4], Yue Wang[1,2,3,4], Danyang Zhang[5], Jianan Tao[1,2,3,4], Zhanning Shen[1,2,3,4], Xuwen Sun[1,2,3,4], Hui Zhou[1,2,3,4], Zixiang Zhu[5], Julian A. Hiscox[6], James P. Stewart[6], Qin Zhao[1,2,3,4*], Yani Sun[1,2,3,4*]

[1] College of Veterinary Medicine, Northwest A&F University, Yangling, 712100, China.

[2] Engineering Research Center of Efficient New Vaccines for Animals, Ministry of Education, Yangling, 712100, China.

[3] Key Laboratory of Ruminant Disease Prevention and Control (West), Ministry of Agriculture and Rural Affairs, Yangling, 712100, China.

[4] Engineering Research Center of Efficient New Vaccines for Animals, Universities of Shaanxi Province, Yangling, 712100, China.

[5] State Key Laboratory for Animal Disease Control and Prevention, College of Veterinary Medicine, Lanzhou University, Lanzhou Veterinary Research Institute, Chinese Academy of Agricultural Sciences, Lanzhou, China.

[6] Department of Infection Biology and Microbiomes, Institute of Infection, Veterinary and Ecological Sciences, University of Liverpool, Liverpool L3 5RF, United Kingdom.

[‡] These authors contributed equally to this work.

[*] Correspondence to Qin Zhao, qinzhao_2004@nwsuaf.edu.cn (QZ); Yani Sun, sunyani@nwsuaf.edu.cn (YNS)

Running title: Nanobody identifies neutralizing epitope on ASFV H240R

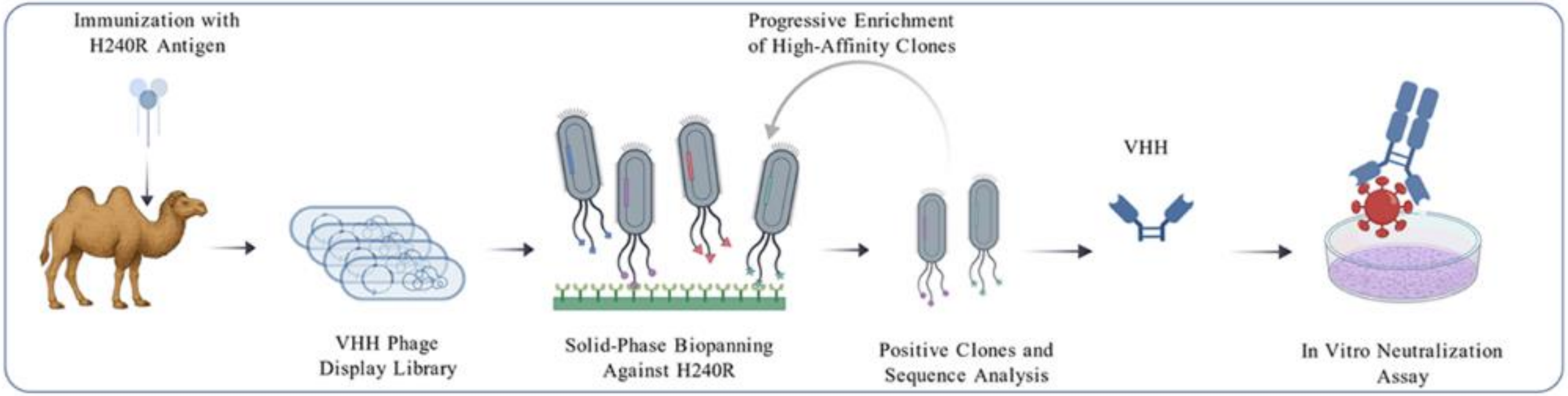

Figure S1 Workflow for the generation of nanobodies against ASFV H240R and neutralization experiments.

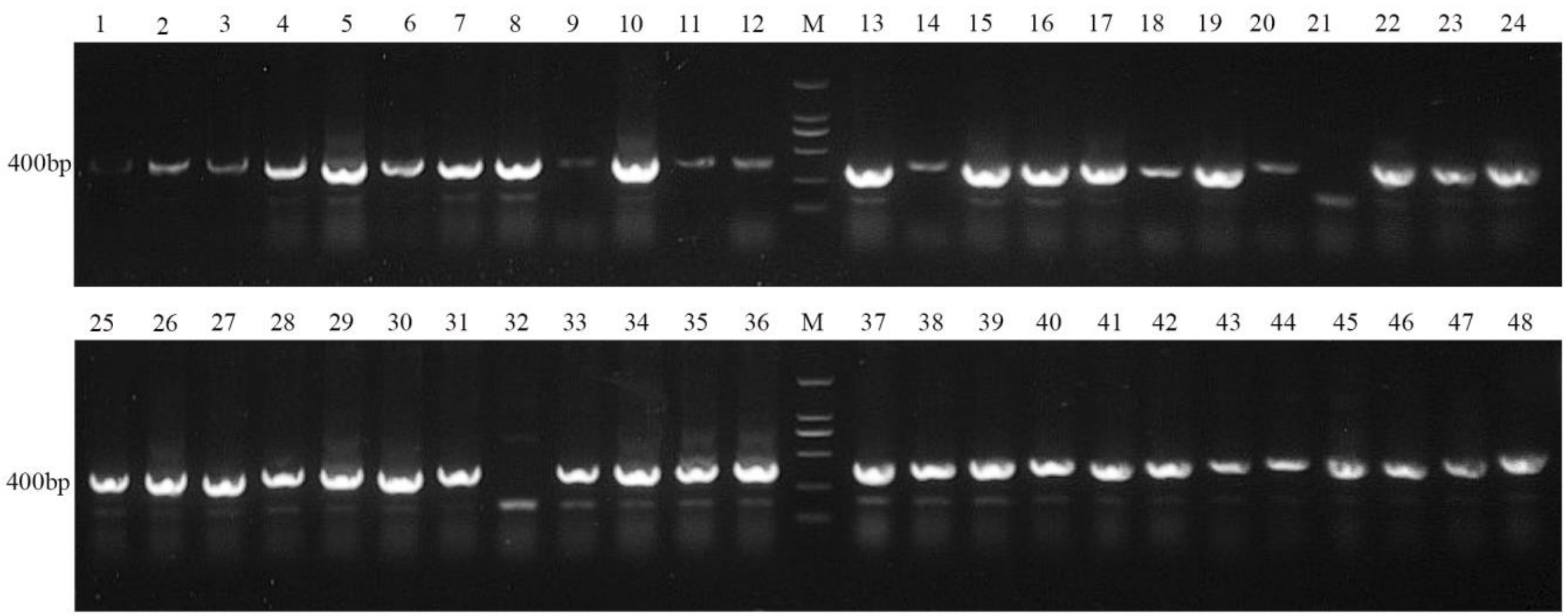

Figure S2 Positive rate of the nanobody library. M: DNA marker (2000, 1000, 750, 500, 250, 100bp). Labels 1-48 denote separate monoclonal colonies.

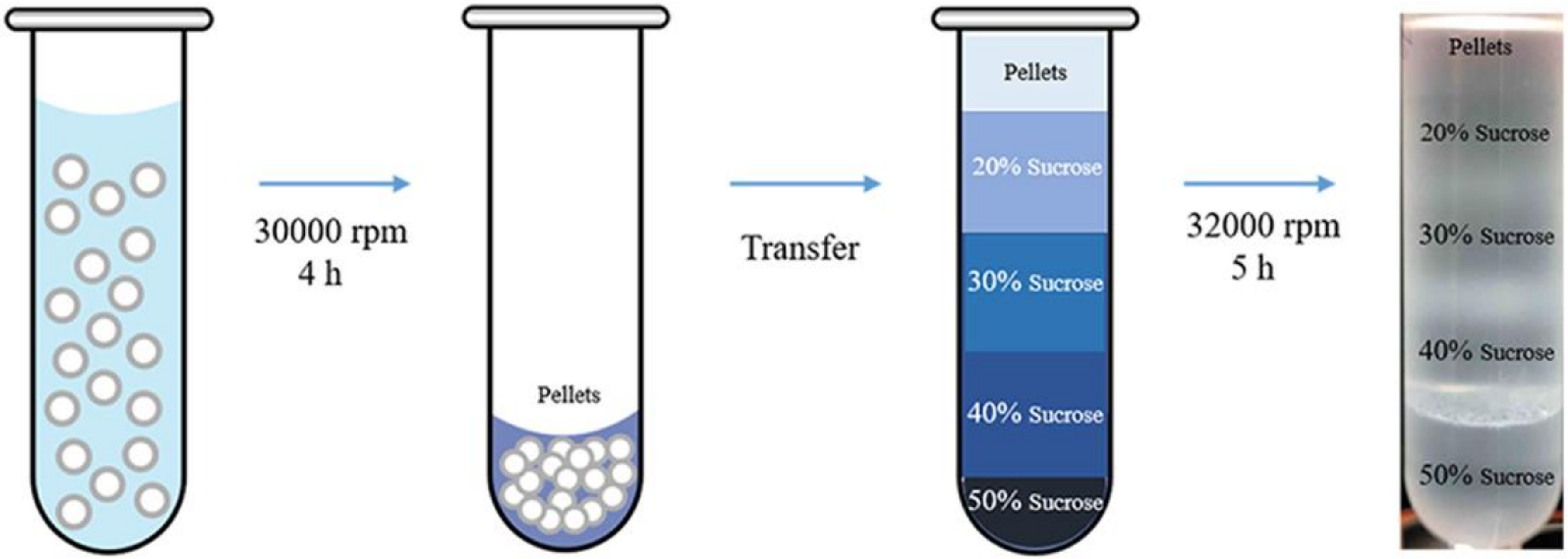

Figure S3 Diagram of sucrose density gradient centrifugation for ASFV virion purification. Virions were collected from the 25%-56% sucrose region of the gradient.

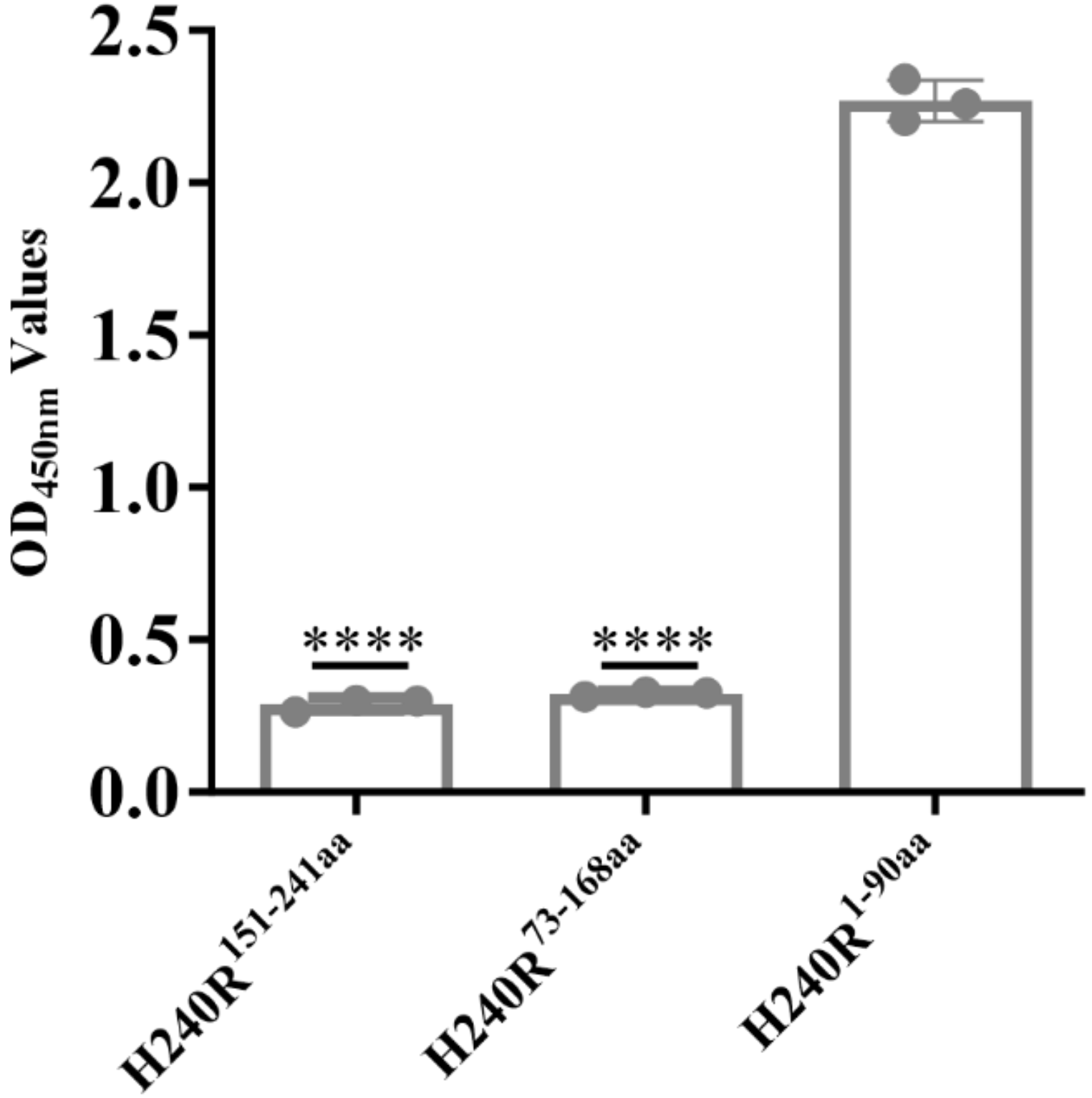

Figure S4 Three recombinant H240R fragments, H240R$^{151\text{-}241aa}$, H240R$^{73\text{-}168aa}$, and H240R$^{1\text{-}90aa}$, were expressed in *E. coli* BL21 (DE3) and used to evaluate their binding activities to H240R-Nb82 by ELISA. For each group, samples were tested in triplicate wells. The data shown are representative of three replicates (Student's t test; ****$P < 0.0001$).

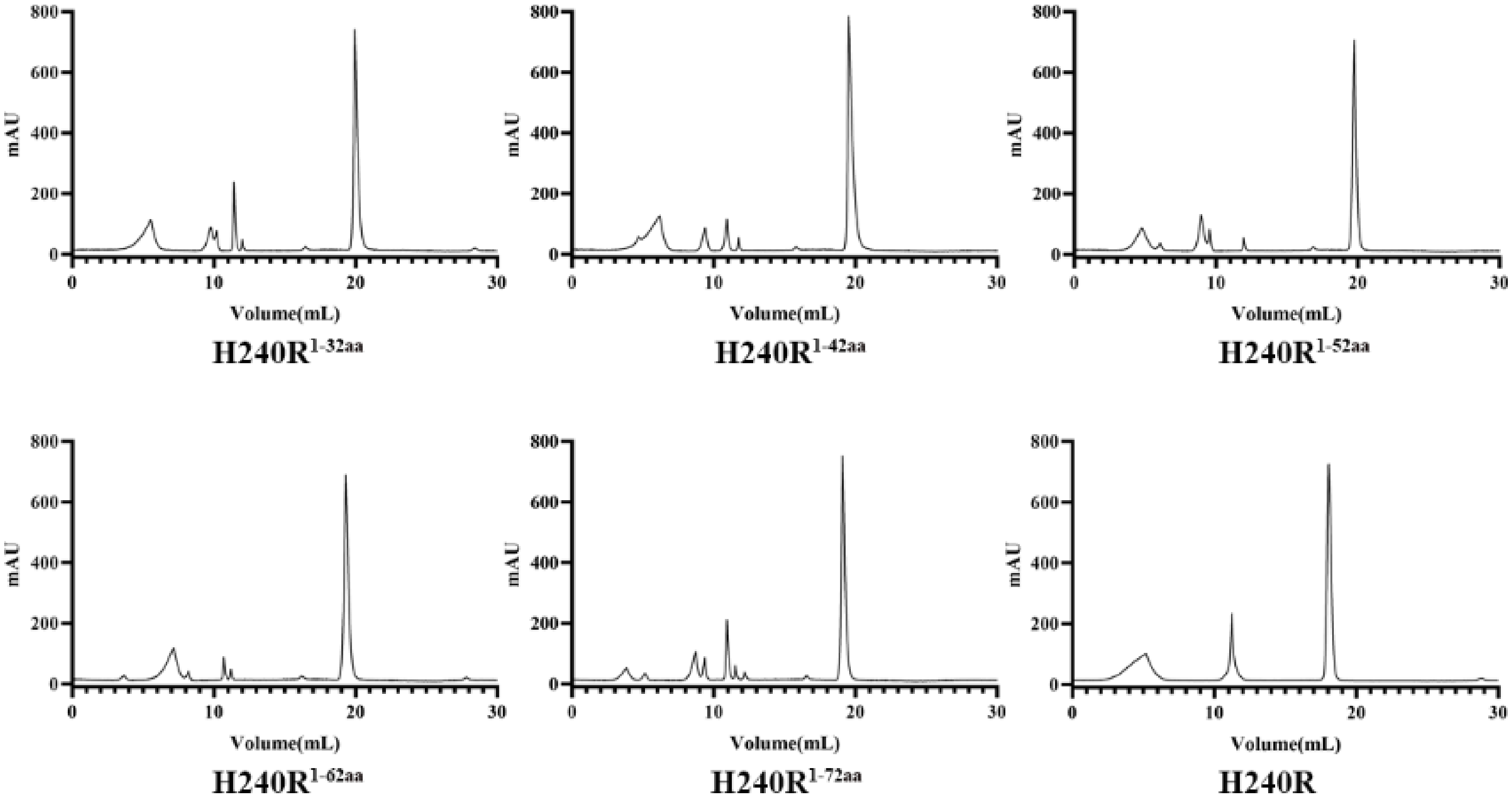

Figure S5 Purified truncated H240R proteins, including H240R$^{1\text{-}32aa}$, H240R$^{1\text{-}42aa}$, H240R$^{1\text{-}52aa}$, H240R$^{1\text{-}62aa}$, H240R$^{1\text{-}72aa}$ and H240R, were analyzed by size-exclusion chromatography to evaluate their homogeneity and aggregation status.

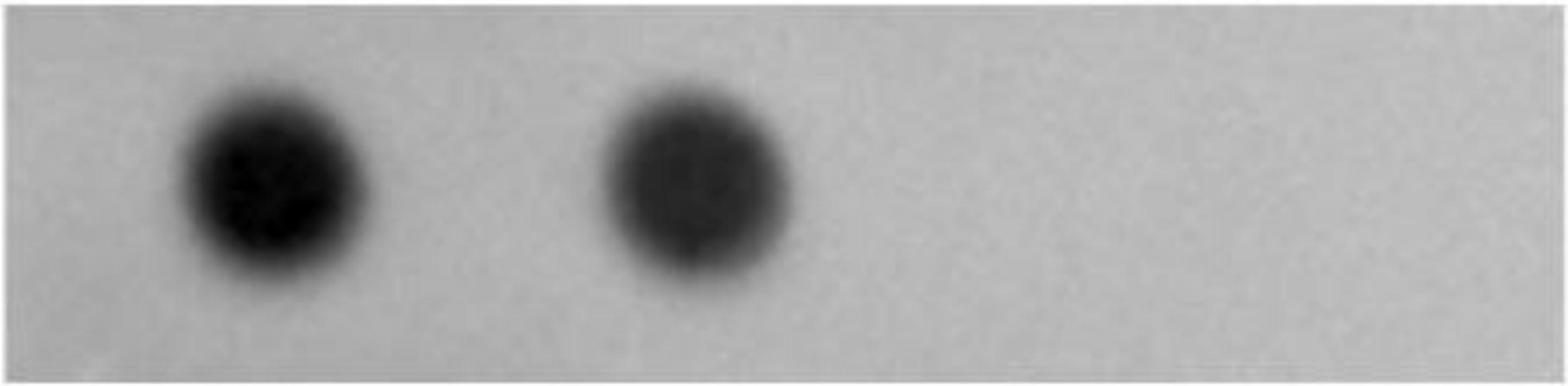

Figure S6 H240R, H240R$^{25-45aa}$ peptide, PRRSV N$^{55-75aa}$ control peptide, and PBS were analyzed by dot blot to evaluate the specific reactivity of H240R-Nb82.

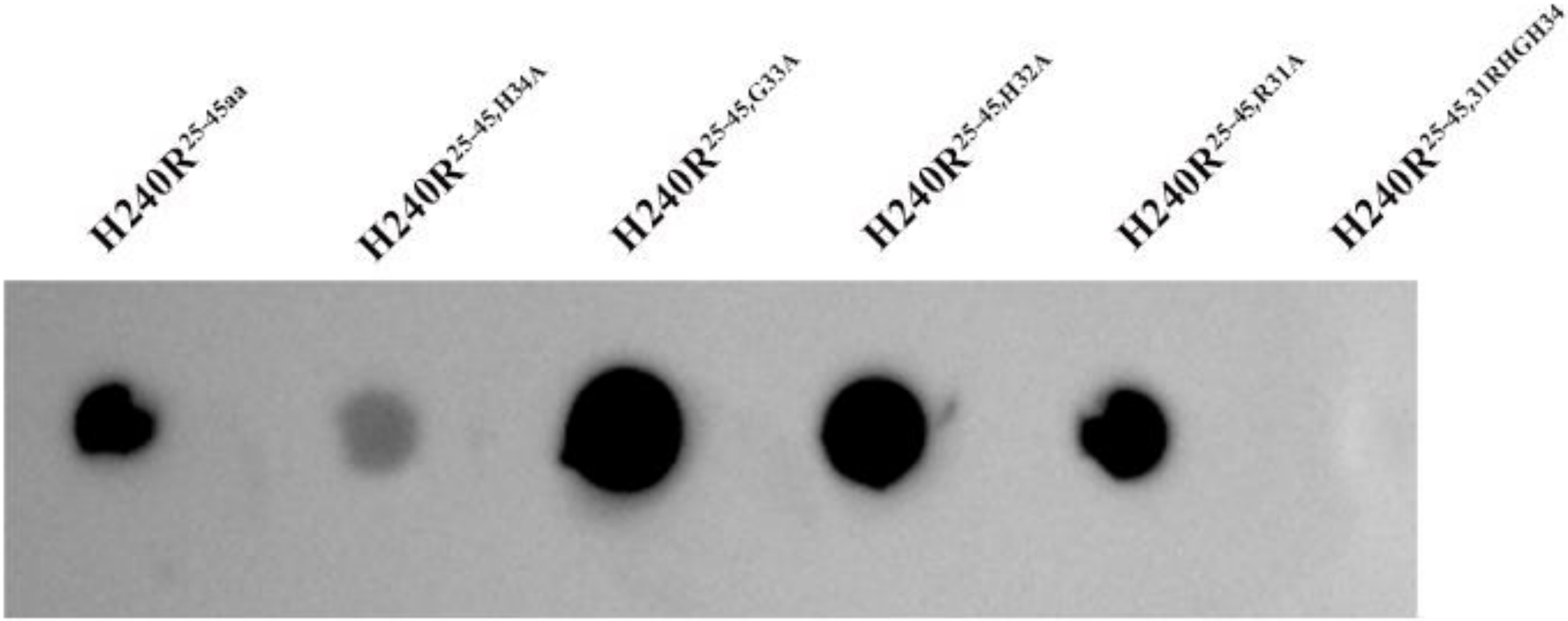

Figure S7 H240R$^{25\text{-}45aa}$ peptide, H240R$^{25\text{-}45aa,\ H34A}$ peptide, H240R$^{25\text{-}45aa,\ G33A}$ peptide, H240R$^{25\text{-}45aa,\ H32A}$ peptide, H240R$^{25\text{-}45aa,\ R31A}$ peptide, H240R$^{25\text{-}45aa,\ 31RHGH34}$ peptide were analyzed by dot blot to evaluate the specific reactivity of H240R-Nb82.

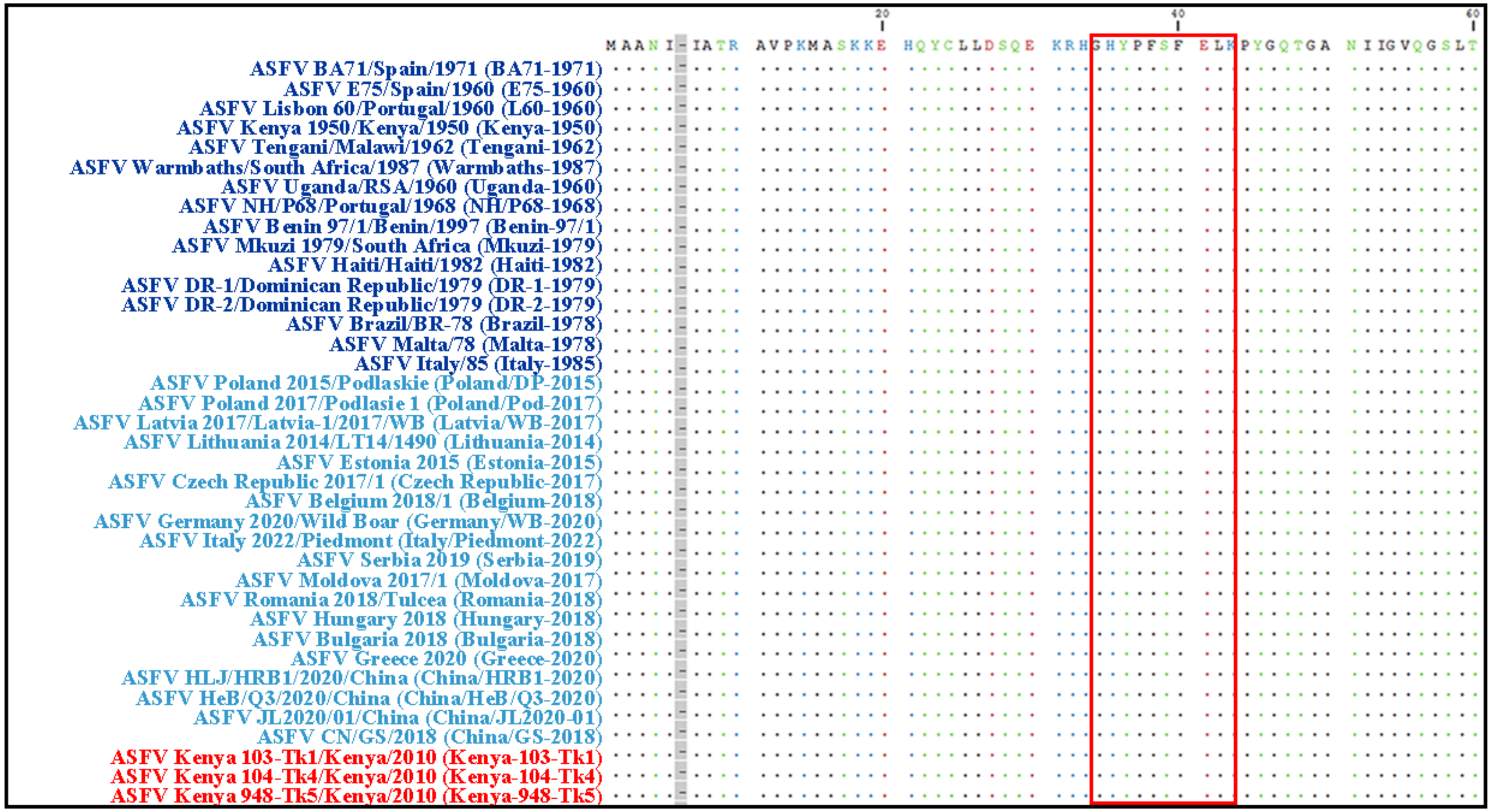

Figure S8 Sequence alignment and conservation analysis of H240R$^{33\text{-}42aa}$ from different ASFV isolates including genotype 1, genotype 2, and recombinant genotype 1 and 2 strains colored dark blue, light blue, and red, respectively.

Table S1 Primers used in this study.

Primer ID	Sequences(5′-3′)
ASFV-p72-F	CGGGTGCGATGATGATTAC
ASFV-p72-R	AGCTGAACCGTTCTGAAGA
ASFV-p72-Probe	ATGCCGATACCACAAGATCAGCCG
CALL001	GTCCTGGCTGCTCTTCTACAAG
CALL002	GGTACGTGCTGTTGAACTGTTCC
VHH-F	CAGGTGCAGCTGCAGGAGTCTGGGGGAG
VHH-R	CTAGTGCGGCCGCTGAGGAGACGGTGACCTGGGT
ASFV-H240R-F	TCGGGATCCAATGGCTGCAAACATTAT
ASFV-H240R-R	GTGCTCGAGCTTAGACGTTTTTAAAG
Trunc -F	CGGGATCCAATGGCTGCAAACATTAT
Trunc1-R	GTGCTCGAGCCCATGACGCT
Trunc2-R	GTGCTCGAGAGGCTTTAATTCAAA
Trunc3-R	GTGCTCGAGTCCTATGATATTTGC
Trunc4-R	GTGCTCGAGTTTGATAACATGGG
Trunc5-R	GTGCTCGAGAAAGGAATCATAAATG
Mut1-F	CCCAGGAAAAGGCTCATGGGCATTATCCCTTTTCA
Mut2-F	CCCAGGAAAAGCGTGCTGGGCATTATCCCTTTTCA
Mut3-F	CCCAGGAAAAGCGTCATGCTCATTATCCCTTTTCA
Mut4-F	CCCAGGAAAAGCGTCATGGGGCTTATCCCTTTTCA
Mut5-F	CCCAGGAAAAGGCTGCTGCTGCTTATCCCTTTTCATTTGAATTAA
